# The descriptive epidemiology and projection of liver cancer in adolescents and young adults: findings from the global burden of disease study 2021

**DOI:** 10.3389/fmed.2025.1690010

**Published:** 2025-12-16

**Authors:** Kai Yang, Meicen Liu, Ke Xu

**Affiliations:** 1 Department of Medical Affairs, National Cancer Center/National Clinical Research Center for Cancer/Cancer Hospital, Chinese Academy of Medical Sciences and Peking Union Medical College, Beijing, China; 2 National Central Cancer Registry, National Cancer Center/National Clinical Research Center for Cancer/Cancer Hospital, Chinese Academy of Medical Sciences and Peking Union Medical College, Beijing, China

**Keywords:** liver cancer, adolescents, young adults, global burden, prediction

## Abstract

**Background:**

The global burden of liver cancer among adolescents and young adults (AYAs, 15–39 years) is an emerging concern. This study analyzes epidemiological trends from 1990 to 2021 and projects future burden to 2030 to inform prevention strategies.

**Methods:**

Using data from the Global Burden of Disease Study 2021, we calculated age-standardized incidence (ASIR) and mortality (ASMR) rates and disability-adjusted life years (DALYs). Joinpoint regression analyzed temporal trends, and a Bayesian age-period-cohort model projected future burden.

**Results:**

From 1990 to 2021, the absolute number of AYA liver cancer cases and deaths increased. However, ASIR peaked in the early 2000s before declining to 0.82 per 100,000 in 2021, with ASMR falling to 0.65 per 100,000. A sharp decline occurred around 2001–2005. The disease burden exhibited significant disparities: males had over double the incidence of females (ASIR 1.22 vs. 0.42). High-burden regions included East Asia and West Africa (e.g., Mongolia, Gambia), while middle-SDI regions carried the highest absolute burden. Projections indicate a continued rise in absolute incident cases (to 45,352) and deaths (to 31,448) by 2030, despite a narrowing of health inequalities.

**Conclusion:**

Although age-standardized rates have declined, the rising absolute burden of AYA liver cancer and persistent disparities highlight a critical need for targeted, equitable prevention and control measures globally.

## Introduction

Hepatocellular carcinoma (HCC), a primary form of liver cancer, continues to be a leading cause of liver-related mortality worldwide, with an estimated 905,700 new cases and 830,200 deaths globally in 2020 (1). The global age-standardized rates (ASRs) for liver cancer are particularly high in regions such as Eastern Asia, Northern Africa, and South-Eastern Asia, where both incidence and mortality rates are significantly elevated (2). Hepatitis B virus (HBV) remains the dominant cause of liver cancer globally, especially in areas with high endemicity such as Southeast Asia and Africa (3). Despite significant advancements in prevention, including the widespread use of the HBV vaccine, antiviral treatments for chronic HBV and hepatitis C (HCV), and improvements in surgical interventions like liver transplantation and resection, liver cancer remains a major cause of morbidity and mortality. While the disease burden has decreased due to these advancements, liver cancer still presents a substantial public health challenge, especially in regions with high HBV and HCV prevalence, as well as those impacted by metabolic risk factors like obesity and diabetes (4).

In recent decades, the age distribution of liver cancer cases has shifted, with an increasing number of younger individuals, being diagnosed with the disease. While liver cancer has traditionally been more prevalent in older populations, studies have shown a rising incidence of HCC among adolescents in several regions (5). This trend is particularly concerning given that chronic HBV infection, which often begins in childhood, can lead to the development of cirrhosis and HCC later in life, underscoring the need for monitoring the long-term effects of early HBV exposure (6). The Global Burden of Disease (GBD) study provides comprehensive data on liver cancer incidence, mortality, and risk factors across 204 countries and regions from 1990 to 2021 (5). One recent analysis revealed a steady increase in the global burden of liver cancer, with both incidence and mortality rates showing significant upward trends, particularly in regions such as Southeast Asia and Eastern Europe (7). Despite advances in HBV vaccination and antiviral treatments, the burden of liver cancer continues to rise due to the increasing prevalence of metabolic risk factors, including obesity, diabetes, and non-alcoholic fatty liver disease (NAFLD), particularly in high socio-demographic index (SDI) regions (8). Moreover, a rising proportion of liver cancer cases in relative younger populations is attributed to the increasing prevalence of metabolic risk factors, alongside the continued importance of viral infections like HBV (9).

While substantial progress has been made in reducing HBV-related liver cancer through vaccination programs, the burden of liver cancer due to non-viral etiologies, such as NAFLD and alcohol consumption, has been rising (9, 10). This shift in etiology highlights the evolving landscape of liver cancer in the younger population, where non-viral liver diseases are emerging as important risk factors (11). Despite extensive research on the global burden of liver cancer, there remains a significant gap in the literature regarding liver cancer specifically in adolescents and young adults (AYAs) aged 15–39 years. Although liver cancer remains a major cause of death in older adults, recent studies have highlighted the rising burden of the disease among younger populations, particularly those with chronic HBV infections acquired in childhood (12). Given the significant morbidity associated with liver cancer and its late-stage diagnosis, early detection and preventive measures for AYAs are critical.

The GBD 2021 database, which provides detailed data on the incidence, mortality, and risk factors of liver cancer across 204 countries and regions, offers a valuable resource for understanding the epidemiology of liver cancer in the AYA population (13). In this study, we used the GBD 2021 data to systematically analyze the epidemiological characteristics of liver cancer in AYAs for the first time, focusing on the rising incidence and mortality trends in this age group. We also employed the Bayesian age-period-cohort (BAPC) model to predict the disease burden from 2022 to 2035, providing projections that will inform public health strategies targeting liver cancer prevention and management in AYAs. This study provides new insights into the rising burden of liver cancer in adolescents and young adults, a population often overlooked in current research. By integrating global and regional data from the GBD 2021 database, we highlight the need for targeted prevention strategies to reduce liver cancer incidence in AYAs, especially those with chronic HBV infections or metabolic risk factors. Our findings will contribute to the development of public health policies aimed at addressing liver cancer in this vulnerable age group, and provide valuable evidence for the prevention and management of liver cancer in young adults worldwide.

## Methods

### Data source and definitions

We obtained data from the Global Burden of Disease (GBD) 2021 study via the Global Health Data Exchange (GHDx)^1^ (14). GBD estimates liver cancer burden in 204 countries and territories using a standardized methodology incorporating national surveys, vital registration systems, cancer registries, and verbal autopsy reports. Liver cancer cases were identified using the International Classification of Diseases (ICD-10) code C22 (15). We extracted liver cancer incidence, mortality, and disability-adjusted life years (DALYs) data stratified by age, sex, location, year, and Socio-demographic Index (SDI) (16). SDI is a composite indicator based on income per capita, education level, and fertility rate, classified into five levels: low, low-middle, middle, high-middle, and high. All data used in mapping and comparative analyses were age-standardized to the GBD global reference population. The data obtained in this study are publicly available from public databases; therefore, no ethical issues are involved.

### Analysis of temporal changes

To assess temporal trends in liver cancer burden from 1990 to 2021, we employed Joinpoint regression analysis using the Joinpoint Regression Program (version 5.0.2, National Cancer Institute, USA) (17). This technique identifies statistically significant changes in trend by fitting a series of joined straight lines on a log scale to the annual rates. The model tests for the presence of “joinpoints,” or points in time where the slope of the trend changes significantly, and selects the optimal number of joinpoints based on the permutation test and the Bayesian Information Criterion (BIC) (18). For each identified segment, the annual percentage change (APC) (19)and corresponding 95% confidence intervals (CIs) were calculated to quantify the magnitude and direction of the trend. The average annual percentage change (AAPC) (20)was also computed over the entire study period to summarize the overall trend. Analyses were stratified by sex, age group, and socio-demographic index (SDI) quintiles to assess subgroup-specific patterns. A *p*-value <0.05 was considered statistically significant for trend changes.

### Subgroup analysis

We analyzed temporal trends in liver cancer burden among individuals aged 15–39 years using three key metrics: age-standardized incidence rate, mortality rate, and disability-adjusted life years (DALYs), comparing estimates from 1990 and 2021. Additionally, Joinpoint regression analysis was performed to evaluate trends in age-standardized incidence, mortality, and DALY rates from 1990 to 2021, stratified by sex. SDI is a composite measure that reflects a country or region’s level of development, incorporating factors such as the fertility rate of women under 25, the average education level of women over 15, and per capita income. It is represented by a value ranging from 0 to 1, with higher values indicating more advanced development. Based on the SDI, countries are categorized into five levels: low, low-middle, middle, high-middle, and high (21).

### Bayesian forecasting of liver cancer burden through 2030

To project the future burden of liver cancer through the year 2030, we adopted a Bayesian age-period-cohort (BAPC) modeling framework, implemented using the R packages BAPC and INLA (Integrated Nested Laplace Approximations).


$$Y_{a,p}∼\text{Poisson}(E_{a,p}\cdot \lambda_{a,p})$$

where *Y_a,p_* denotes the number of cases in age group *a* and period *p*, *Ea,p* ​is the corresponding population at risk (offset), and *λa,p* ​is the underlying rate. The log-rate is modeled additively as:


$$\log(\lambda_{a,p})=\alpha_{a}+\beta_{p}+\gamma_{c},\text{with}\;c=p-a$$

Here, α_a_​, β*_p_* and γ*_c_* represent age, period, and cohort effects, respectively. To ensure smoothness, each effect was assigned a second-order random walk prior (RW2), which penalizes sudden fluctuations in adjacent time units.

Model inference was performed using the Integrated Nested Laplace Approximation (INLA), implemented via the INLA and BAPC R packages. INLA provides a computationally efficient alternative to Markov chain Monte Carlo (MCMC) by applying deterministic approximations to the marginal posterior distributions. Specifically, the posterior of the hyperparameters *θ* is approximated as:


p(θ∣y)≈p∼(θ∣y)

where p ~ (θ∣y) is obtained through a series of nested Laplace approximations over the latent Gaussian field. This approach allows for fast, accurate, and scalable Bayesian inference in complex hierarchical models. The BAPC model incorporates three temporal dimensions—age, calendar year (period), and birth cohort—to estimate future trends while accounting for the inherent autocorrelation and uncertainty in each component. This approach is particularly suited for forecasting disease burden in young populations, where cohort effects may be pronounced. The model was calibrated using historical data from 1990 to 2021, and posterior estimates were generated using Bayesian inference with non-informative priors. Forecasts were performed separately for incidence, mortality, and disability-adjusted life years (DALYs) in the 15–39 age group and disaggregated by sex and SDI quintile. Disability-Adjusted Life Years (DALYs) (22)were used to quantify the overall burden of liver cancer. DALYs integrate the years of life lost due to premature mortality (YLLs) and the years lived with disability (YLDs), offering a comprehensive metric that reflects both fatal and non-fatal outcomes.

The calculation follows the standard Global Burden of Disease (GBD) methodology:


DALYs=YLLs+YLDs

### Uncertainty intervals

All point estimates are presented with 95% uncertainty intervals (UIs) (23), which reflect the 2.5th and 97.5th percentiles of 1,000 posterior draws from the Bayesian models used in GBD estimation. These UIs quantify the statistical uncertainty around each estimate rather than relying on conventional confidence intervals.

### Statistical analysis

All data processing and statistical analyses were conducted using R version 4.3.2. Initial data cleaning, wrangling, and transformation were performed using the tidyverse suite, including dplyr, tidyr, and readr. Visualization of trends and forecasts was performed using ggplot2, which was used to construct line graphs, dot plots, and choropleth maps. All visualizations incorporated measures of uncertainty, derived from the 2.5th and 97.5th percentiles of the Global Burden of Disease (GBD) estimates or Bayesian posterior distributions, where appropriate. To ensure reproducibility, all code and analytical scripts were version-controlled and documented. A *p*-value of <0.05 indicated statistical significance.

## Results

### Liver cancer incidence and mortality among adolescents and young adults (AYAs), 1990–2021

According to Table 1, the global burden of liver cancer among adolescents and young adults (AYAs) aged 15–39 years remained considerable between 1990 and 2021. In 1990, there were an estimated 19,864 incident cases of liver cancer (95% UI: 17,389 –22,886), corresponding to an age-specific incidence rate of 0.91 per 100,000 (95% UI: 0.79–1.04). The number of deaths reached 17,748 (95% UI: 15,532 –20,436), with a mortality rate of 0.81 per 100,000 (95% UI: 0.71–0.93). By 2021, incident cases increased to 24,348 (95% UI: 21,491 –28,273), while the incidence rate showed a mild decline to 0.82 per 100,000 (95% UI: 0.72–0.95). Meanwhile, liver cancer–related deaths rose to 19,270 (95% UI: 17,037 –22,393), with the mortality rate decreasing to 0.65 per 100,000 (95% UI: 0.57–0.75). Across regions, the highest liver cancer incidence and mortality in AYAs were observed in East Asia in both years (1990 incidence rate: 2.21 per 100,000 [1.84–2.64]; deaths: 1.99 per 100,000 [1.65–2.37]; 2021 incidence: 2.54 [2.01–3.25]; deaths: 1.85 [1.47–2.38]). Central Asia, Southern Sub-Saharan Africa, and High-middle SDI regions also maintained relatively high rates. Conversely, Southern Latin America exhibited the lowest incidence and mortality rates during the study period.

**Table 1:** Incidence and deaths of liver cancer in adolescents and young adults by SDI, and region in 1990 and 2021.

Location	1990		1990		2021		2021	
	Incidence rate per 100,000 No. (95% UI)	Incidence cases (95% UI)	Death rate per 100,000 No. (95% UI)	Death cases (95% UI)	Incidence rate per 100,000 No. (95% UI)	Incidence cases (95% UI)	Death rate per 100,000 No. (95% UI)	Death cases (95% UI)
Global	0.91 (0.79–1.04)	19863.66 (17389.28–22885.63)	0.81 (0.71–0.93)	17748.11 (15531.71–20436.12)	0.82 (0.72–0.95)	24348.06 (21491.13–28273.11)	0.65 (0.57–0.75)	19270.22 (17036.81–22393.10)
High SDI	0.55 (0.50–0.62)	1911.08 (1723.54–2146.93)	0.43 (0.39–0.49)	1499.03 (1343.24–1694.78)	0.54 (0.51–0.58)	1911.47 (1805.61–2035.15)	0.32 (0.30–0.34)	1122.97 (1060.27–1191.16)
High-income Asia Pacific	1.31 (1.04–1.67)	887.08 (698.69–1127.76)	1.04 (0.80–1.35)	704.45 (540.77–910.35)	0.79 (0.65–0.98)	399.11 (326.51–493.50)	0.41 (0.34–0.51)	205.76 (169.91–258.74)
High-income North America	0.29 (0.28–0.30)	325.55 (316.88–334.33)	0.18 (0.18–0.19)	207.65 (203.51–211.82)	0.50 (0.48–0.53)	618.05 (589.23–648.16)	0.26 (0.25–0.27)	322.40 (309.51–336.12)
High-middle SDI	1.22 (1.03–1.48)	5532.12 (4657.17–6689.46)	1.09 (0.92–1.33)	4941.57 (4151.35–6003.75)	1.22 (0.98–1.54)	5386.91 (4295.19–6784.88)	0.90 (0.72–1.13)	3960.24 (3172.53–4987.75)
Low SDI	0.68 (0.48–0.97)	1250.29 (885.05–1780.80)	0.63 (0.45–0.89)	1162.82 (823.09–1648.82)	0.61 (0.48–0.81)	2719.06 (2136.52–3635.80)	0.56 (0.44–0.75)	2512.13 (1963.58–3374.09)
Low-middle SDI	0.42 (0.36–0.53)	1896.75 (1614.40–2416.99)	0.39 (0.33–0.49)	1756.61 (1495.88–2232.82)	0.48 (0.41–0.57)	3837.57 (3290.94–4538.98)	0.43 (0.37–0.52)	3482.89 (2979.80–4135.12)
Middle SDI	1.23 (1.07–1.43)	9266.33 (8084.58–10766.85)	1.11 (0.97–1.29)	8381.64 (7309.34–9702.61)	1.13 (0.96–1.39)	10484.52 (8891.99–12866.82)	0.88 (0.75–1.07)	8184.62 (6945.89–9954.80)
Andean Latin America	0.34 (0.29–0.42)	52.47 (44.16–64.37)	0.32 (0.27–0.39)	48.95 (41.41–59.97)	0.29 (0.22–0.37)	77.48 (59.29–101.22)	0.25 (0.19–0.33)	68.70 (52.65–89.05)
Australasia	0.24 (0.22–0.26)	19.54 (17.54–21.58)	0.19 (0.17–0.20)	15.10 (13.65–16.68)	0.59 (0.51–0.67)	61.31 (53.49–70.68)	0.36 (0.32–0.41)	37.64 (33.25–43.05)
Caribbean	0.19 (0.16–0.22)	27.70 (24.27–32.00)	0.17 (0.15–0.20)	25.07 (21.96–29.00)	0.19 (0.15–0.23)	34.51 (27.45–42.37)	0.17 (0.13–0.21)	30.36 (23.83–37.50)
Central Asia	0.71 (0.63–0.81)	203.18 (180.32–230.73)	0.66 (0.59–0.75)	188.27 (166.98–213.48)	0.61 (0.50–0.72)	226.71 (187. – 268.43)	0.55 (0.46–0.66)	207.10 (171.19–245.73)
Central Europe	0.28 (0.25–0.30)	129.86 (118.53–142.56)	0.25 (0.23–0.28)	117.96 (107.77–129.46)	0.19 (0.17–0.21)	67.10 (59.89–75.04)	0.17 (0.15–0.19)	58.49 (51.96–65.28)
Central Latin America	0.20 (0.19–0.20)	133.49 (129.75–138.05)	0.18 (0.18–0.19)	123.02 (119.68–127.15)	0.23 (0.20–0.25)	227.74 (204.73–251.33)	0.20 (0.18–0.22)	201.70 (180.72–222.92)
Central Sub-Saharan Africa	0.64 (0.28–1.48)	132.17 (57.99–307.06)	0.59 (0.26–1.37)	123.08 (53.82–284.53)	0.45 (0.20–1.07)	244.55 (106.48–580.31)	0.42 (0.18–1.04)	226.73 (97.83–560.60)
East Asia	2.21 (1.84–2.64)	12515.90 (10403.11–14950.78)	1.99 (1.65–2.37)	11235.41 (9357.86–13417.81)	2.54 (2.01–3.25)	12148.88 (9606.79–15553.45)	1.85 (1.47–2.38)	8883.85 (7029.84–11422.96)
Eastern Europe	0.21 (0.20–0.22)	182.35 (173.16–191.78)	0.19 (0.18–0.20)	165.76 (157.78–174.36)	0.30 (0.27–0.32)	195.28 (180.58–209.91)	0.26 (0.24–0.28)	170.15 (157.29–182.78)
Eastern Sub-Saharan Africa	0.53 (0.43–0.69)	376.13 (301.62–487.88)	0.50 (0.40–0.64)	351.48 (282.10–452.94)	0.50 (0.36–0.70)	870.94 (630.92–1218.90)	0.46 (0.33–0.65)	806.47 (581.95–1141.00)
North Africa and Middle East	0.35 (0.29–0.45)	466.48 (381.63–600.43)	0.32 (0.26–0.41)	429.25 (351.47–553.87)	0.41 (0.35–0.48)	1041.47 (885.34–1212.00)	0.36 (0.30–0.41)	907.53 (769.55–1055.12)
Oceania	0.51 (0.29–1.02)	13.57 (7.70–27.02)	0.47 (0.27–0.94)	12.49 (7.12–24.93)	0.43 (0.27–0.78)	24.36 (15.35–43.98)	0.39 (0.25–0.71)	22.09 (13.82–40.01)
South Asia	0.23 (0.20–0.27)	986.27 (873.35–1161.16)	0.21 (0.19–0.25)	914.51 (809.93–1072.66)	0.32 (0.27–0.38)	2503.08 (2161.53–2999.50)	0.29 (0.25–0.34)	2265.34 (1957.59–2712.57)
Southeast Asia	0.82 (0.71–0.96)	1624.22 (1408.47–1899.89)	0.76 (0.65–0.89)	1489.61 (1290.16–1746.27)	0.79 (0.64–1.05)	2189.89 (1782.72–2922.32)	0.69 (0.56–0.92)	1905.02 (1547.15–2545.24)
Southern Latin America	0.05 (0.05–0.06)	10.28 (8.69–12.07)	0.05 (0.04–0.06)	9.13 (7.71–10.71)	0.11 (0.10–0.13)	29.60 (25.88–33.37)	0.09 (0.08–0.11)	24.38 (21.32–27.51)
Southern Sub-Saharan Africa	1.18 (0.76–1.79)	255.17 (164.58–385.93)	1.08 (0.70–1.64)	233.23 (150.49–354.12)	1.54 (1.22–1.91)	522.57 (416.86–649.06)	1.38 (1.11–1.73)	471.32 (377.29–587.72)
Tropical Latin America	0.17 (0.17–0.18)	111.37 (106.72–116.20)	0.16 (0.15–0.17)	102.22 (98.09–106.41)	0.17 (0.16–0.18)	151.47 (143.32–159.77)	0.15 (0.14–0.16)	134.06 (126.97–141.40)
Western Europe	0.25 (0.24–0.26)	356.85 (343.01–371.03)	0.19 (0.18–0.20)	273.67 (263.38–284.63)	0.41 (0.38–0.43)	526.01 (499.53–555.59)	0.24 (0.23–0.25)	306.86 (292.48–320.86)
Western Sub-Saharan Africa	1.47 (0.94–2.21)	1054.04 (671.43–1584.92)	1.37 (0.87–2.05)	977.81 (623.31–1469.47)	1.14 (0.88–1.44)	2187.93 (1677.07–2743.96)	1.05 (0.81–1.33)	2014.27 (1543.64–2534.55)

UI, uncertainty intervals.

From a socioeconomic perspective, the middle SDI region carried the greatest absolute burden in 2021, with 10,485 incident cases (95% UI: 8,892 –12,867) and 8,185 deaths cases (95% UI: 6,946 –9,955). Although the high SDI region had lower rates (incidence: 0.54 [0.51–0.58]; mortality: 0.32 [0.30–0.34]), it still accounted for a notable share of the global total due to population size. In contrast, both low SDI and low-middle SDI regions experienced increasing absolute numbers but continued declines in age-standardized rates, reflecting persistent disparities in early detection, treatment accessibility, and HBV vaccination coverage.

### Global changes in incidence, mortality, and DALYs among individuals aged 15–39 years

The age-standardized incidence rate (ASIR) of liver cancer among adolescents and young adults (15–39 years) varied markedly across countries between 1990 and 2021 (Supplementary Table S1 and Supplementary Figures S1, S2). In 1990, the highest ASIRs were reported in Mongolia (4.44 [2.89–6.59] per 100,000), Guinea-Bissau (4.68 [1.92–7.49]), Gambia (3.52 [2.32–5.05]), Benin (2.65 [1.35–4.20]), and China (2.22 [1.84–2.66]), indicating a heavy disease burden in parts of East Asia and West Africa. In contrast, the lowest ASIRs (< 0.10 per 100,000) were found in Morocco (0.04 [0.03–0.05]), Argentina (0.04 [0.03–0.05]), Chile (0.07 [0.06–0.09]), Poland (0.06 [0.05–0.06]), Jamaica (0.06 [0.05–0.08]), Uruguay (0.06 [0.05–0.07]). By 2021, the ASIR showed heterogeneous changes. The highest rates persisted in Mongolia (5.05 [3.47–7.28]), Gambia (4.54 [2.77–7.38]), Tonga (2.51 [1.50–4.24]), China (2.57 [2.02–3.30]), and Thailand (2.07 [1.36–3.00]), with several African countries such as Guinea (2.67 [1.69–4.00]) and Cabo Verde (2.27 [1.57–3.22]) also remaining elevated. Conversely, low ASIRs (<0.10 per 100,000) were observed in Morocco (0.05 [0.03–0.08]), Kuwait (0.08 [0.06–0.10]), Mauritius (0.06 [0.06–0.07]). Some high-income regions experienced noticeable increases. The United Kingdom rose from 0.19 (0.18–0.19) in 1990 to 0.74 (0.71–0.78) in 2021, Australia from 0.22 (0.19–0.25) to 0.55 (0.47–0.65), Germany from 0.17 (0.14–0.19) to 0.31 (0.27–0.37), and the United States of America from 0.29 (0.28–0.30) to 0.50 (0.48–0.53). The global maps (Supplementary Figure S1 for 1990 and Supplementary Figure S2 for 2021) visualize these shifts: nations with ASIRs exceeding 1.0 per 100,000 were mainly clustered in East Asia, Pacific islands, and West Africa, while large areas of Europe and the Americas remained at comparatively low levels throughout the study period.

The age-standardized mortality rate (ASMR) of liver cancer among adolescents and young adults aged 15–39 years varied widely across countries between 1990 and 2021 (Supplementary Table S2 and Supplementary Figures S3, S4). In 1990, the highest ASMRs were observed in Mauritania (4.65 [0.95–9.69] per 100,000), Guinea-Bissau (4.35 [1.79–6.99]) and Mongolia (4.17 [2.71–6.13]). In contrast, very low mortality (<0.10 per 100,000) occurred in Uruguay (0.05 [0.04–0.06]), Poland (0.05 [0.05–0.06]), and Malta (0.08 [0.07–0.09]), reflecting minimal disease burden. By 2021, the pattern remained heterogeneous. The highest ASMRs persisted in Mongolia (4.62 [3.19–6.60]), Gambia (4.16 [2.56–6.77]) and Tonga (2.22 [1.32–3.70]), while Lesotho (2.48 [0.81–7.32]) and Cabo Verde (2.02 [1.40–2.84]) also recorded elevated mortality. Conversely, the lowest ASMRs were still found in Mauritius (0.05 [0.05–0.06]), Malta (0.14 [0.12–0.17]), and Poland (0.12 [0.11–0.13]). Spatially, the 1990 map (Supplementary Figure S3) shows mortality “hotspots” concentrated in West Africa, East Asia, and the Pacific islands, while the 2021 map (Supplementary Figure S4) indicates partial reductions in East Asia but sustained high burdens in several African nations.

The age-standardized DALY rate of liver cancer among adolescents and young adults (15–39 years) exhibited pronounced national variation from 1990 to 2021 (Supplementary Table S3 and Supplementary Figures S5, S6). In 1990, the highest DALY rates were recorded in Mauritania (273.85 [56.64–576.86] per 100,000), Guinea-Bissau (257.54 [108.65–413.54]) and Mongolia (250.93 [165.20–367.17]), reflecting extreme disease burdens in parts of West Africa and East Asia. Conversely, the lowest DALY rates were found in Portugal (11.16 [9.62–12.88]), Romania (8.64 [6.88–10.51]), Mexico (7.19 [7.05–7.34]) and Algeria (7.92 [5.93–10.48]). By 2021, the highest DALY levels persisted in Mongolia (266.94 [186.28–378.61]), Gambia (246.05 [152.11–400.21]) and Tonga (127.58 [76.83–208.74]), while Lesotho (142.67 [47.40–420.02]) and Liberia (142.68 [80.83–218.79]) also showed elevated DALY burdens. Spatially, Supplementary Figure S5 highlights DALY “hotspots” across West Africa, East Asia, and selected Pacific islands, whereas Supplementary Figure S6 shows persistence of these high-burden clusters alongside growing intermediate burdens in several developed countries.

### Sex-specific and SDI-stratified temporal trends in disease burden (1990–2021)

**Figure 1:**
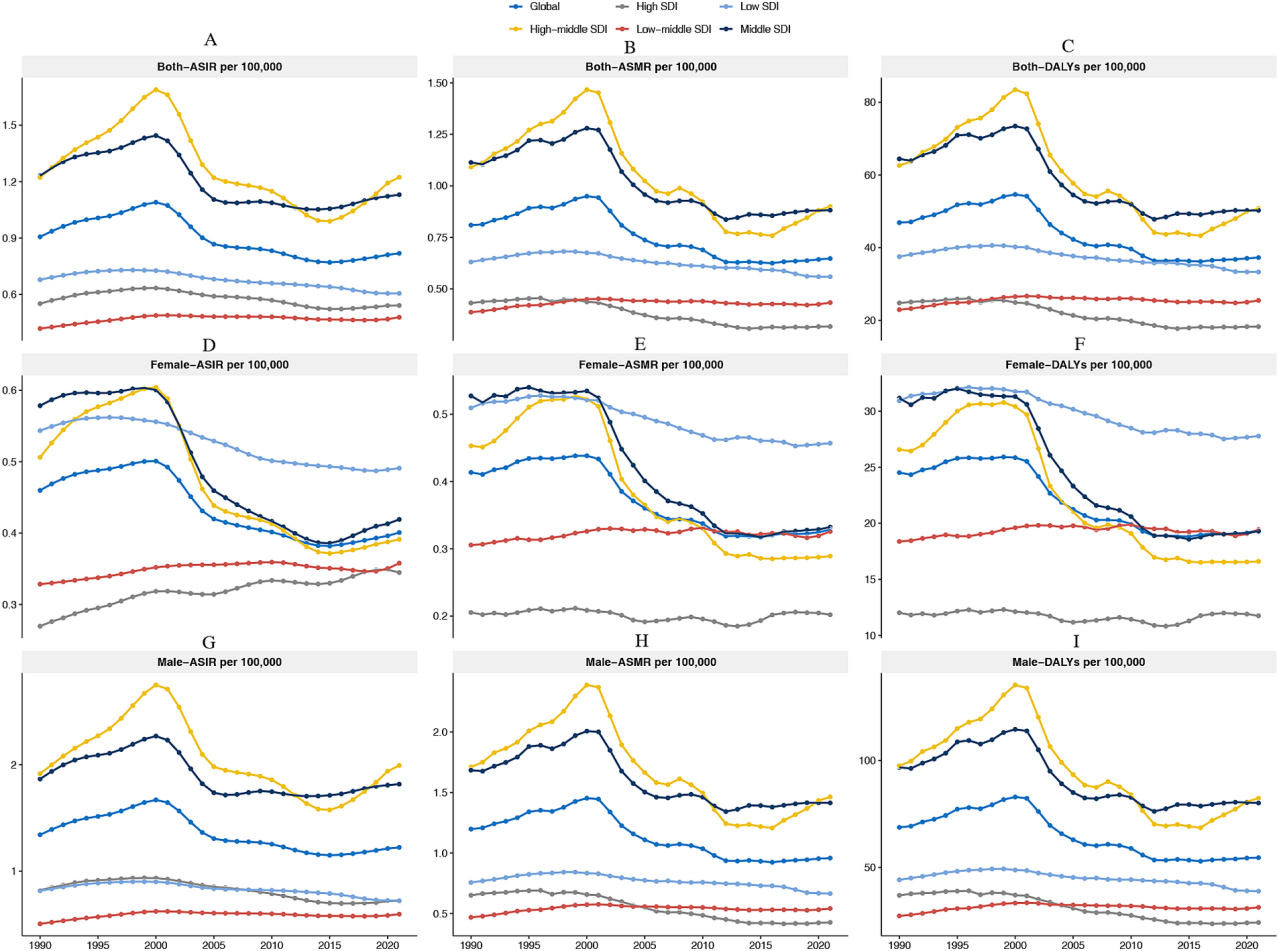
Temporal trends in age-standardized incidence rates (ASIR), mortality rates (ASMR), and disability-adjusted life years (DALYs) by sex and Socio-demographic Index (SDI), 1990–2021. The first row presents trends for both sexes: ASIR **(A)**, ASMR **(B)**, and age-standardized DALYs **(C)**. The second row shows female-specific trends: ASIR **(D)**, ASMR **(E)**, and age-standardized DALYs **(F)**. The third row illustrates male-specific trends: ASIR **(G)**, ASMR **(H)**, and age-standardized DALYs **(I)**. Colors represent different SDI levels: global, high, high-middle, middle, low-middle, and low.

Figure 1 illustrates the sex-specific and SDI-stratified temporal trends in liver cancer burden among adolescents and young adults (AYAs) aged 15–39 years between 1990 and 2021. Globally, the age-standardized incidence rate (ASIR) of liver cancer showed a moderate increase from 0.91 per 100,000 (95% UI: 0.79–1.04) in 1990 to a peak of 1.05 per 100,000 (95% UI: 0.93–1.19) in the early 2000s, before declining to 0.82 per 100,000 (95% UI: 0.72–0.95) in 2021. Similarly, the age-standardized mortality rate (ASMR) increased from 0.81 per 100,000 (95% UI: 0.71–0.93) in 1990 to a peak in the early 2000s, and then decreased to 0.65 per 100,000 (95% UI: 0.57–0.75) by 2021. The disability-adjusted life years (DALYs) followed a similar trajectory, with an initial increase and then a decline, from 46.89 per 100,000 (95% UI: 44.39–49.80) in 1990 to 37.27 per 100,000 (95% UI: 33.86–41.03) in 2021 (Supplementary Table S4).

When analyzed by sex, males continued to experience a higher disease burden compared to females. In 2021, the ASIR for males was 1.22 per 100,000 (95% UI: 1.04–1.40), while for females it was significantly lower at 0.42 per 100,000 (95% UI: 0.34–0.50). The ASMR for males in 2021 was 0.96 per 100,000 (95% UI: 0.87–1.06), compared to 0.33 per 100,000 (95% UI: 0.27–0.39) for females. Similarly, DALYs in males were 54.63 per 100,000 (95% UI: 49.56–59.98), while for females, DALYs were 19.38 per 100,000 (95% UI: 17.16–21.58). Stratifying by socio-demographic index (SDI), countries in the high SDI quintile continued to have the lowest rates, although the incidence rate slightly increased over the past two decades. In 2021, the ASIR for high-SDI countries was 0.54 per 100,000 (95% UI: 0.51–0.58), and the ASMR was 0.32 per 100,000 (95% UI: 0.30–0.34) (Supplementary Table S4).

### Temporal trend analysis of age-standardized rates using joinpoint regression

**Figure 2:**
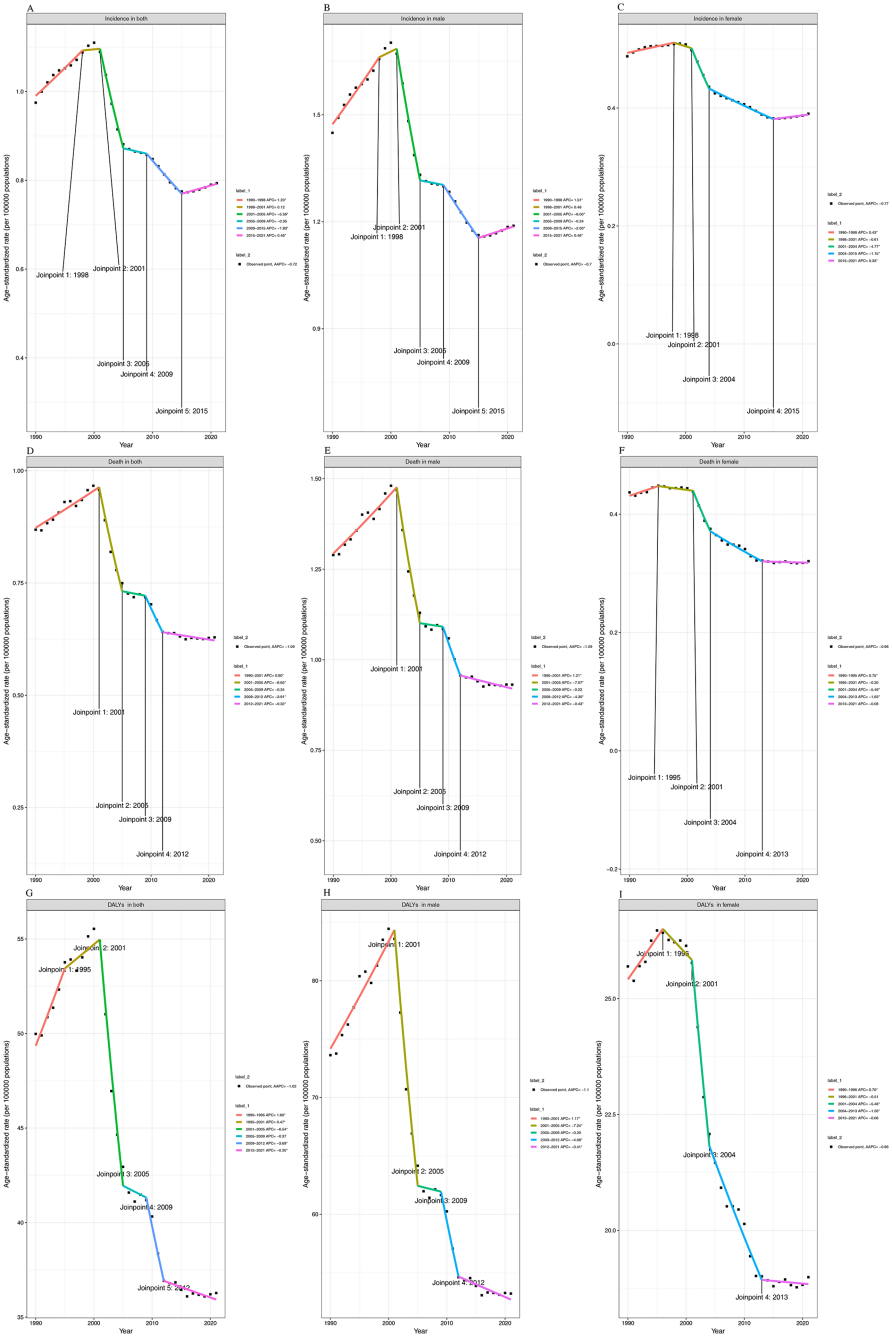
Temporal Trends in Age-Standardized Rates with Joinpoint Regression Analysis, 1990–2021. The first row presents trends in age-standardized incidence rates (ASIR) for both sexes **(A)**, males **(B)**, and females **(C)**. The second row illustrates age-standardized mortality rates (ASMR) for both sexes **(D)**, males **(E)**, and females **(F)**. The third row shows trends in age-standardized disability-adjusted life years (DALYs) for both sexes **(G)**, males **(H)**, and females **(I)**. All panels include joinpoints highlighting significant changes in trends over time. The vertical axis represents rates per 100,000 population.

For the age-standardized incidence rate (ASIR) in the total population, the overall trend demonstrated a significant decline, with an average annual percent change (AAPC) of −0.72%. The trend could be segmented into six distinct phases: a modest increase from 1990 to 1998 (APC = 1.23%, *p* < 0.05), a stable period between 1998 and 2001 (APC = 0.12%), followed by a sharp decline from 2001 to 2005 (APC = −5.56%, *p* < 0.05). From 2005 to 2009, the trend plateaued slightly (APC = −0.35%), continued to decline between 2009 and 2015 (APC = −1.83%, *p* < 0.05), and rebounded mildly from 2015 to 2021 (APC = 0.48%, *p* < 0.05) (Figures 2A–C). Among males, a similar segmented trend was observed, with an AAPC of −0.70%. The ASIR increased from 1990 to 1998 (APC = 1.51%, *p* < 0.05), remained stable from 1998 to 2001 (APC = 0.48%), and declined significantly between 2001 and 2005 (APC = −6.00%, *p* < 0.05). Subsequent changes were minor until a mild resurgence after 2015 (APC = 0.46%, *p* < 0.05). In females, the incidence declined more gradually (AAPC = −0.77%), with notable declines during 2001–2004 (APC = −4.77%, *p* < 0.05) and 2004–2015 (APC = −1.15%, *p* < 0.05) (Supplementary Tables S5, S6). The ASMR declined steadily during the study period, with an AAPC of −1.09% in both sexes. The mortality trend increased slightly from 1990 to 2001 (APC = 0.90%, *p* < 0.05), then dropped sharply between 2001 and 2005 (APC = −6.65%, *p* < 0.05), and continued to decline, though at a slower rate thereafter. The mortality reduction was more pronounced in males, particularly during 2001–2005 (APC = −7.07%, *p* < 0.05), whereas females showed a less steep decline during similar periods (e.g., APC = −5.49% in 2001–2004, *p* < 0.05), with an overall AAPC of −0.98% (Figures 2D–F and Supplementary Tables S7, S8). For DALYs, the age-standardized rate declined with an AAPC of −1.02% in the total population. After a brief rise between 1990 and 1995 (APC = 1.60%, *p* < 0.05), the burden remained relatively stable until 2001, followed by a substantial drop between 2001 and 2005 (APC = −6.54%, *p* < 0.05) (Supplementary Tables S9, S10). The decline continued through 2021, though with attenuated slope. In males, the AAPC was −1.10%, with the steepest drop again occurring from 2001 to 2005 (APC = −7.24%, *p* < 0.05). Females exhibited more moderate reductions, with an AAPC of −0.96% (Figures 2G–I).

### Projected trends in incident cases and deaths of disease among young people (2022–2030)

**Figure 3:**
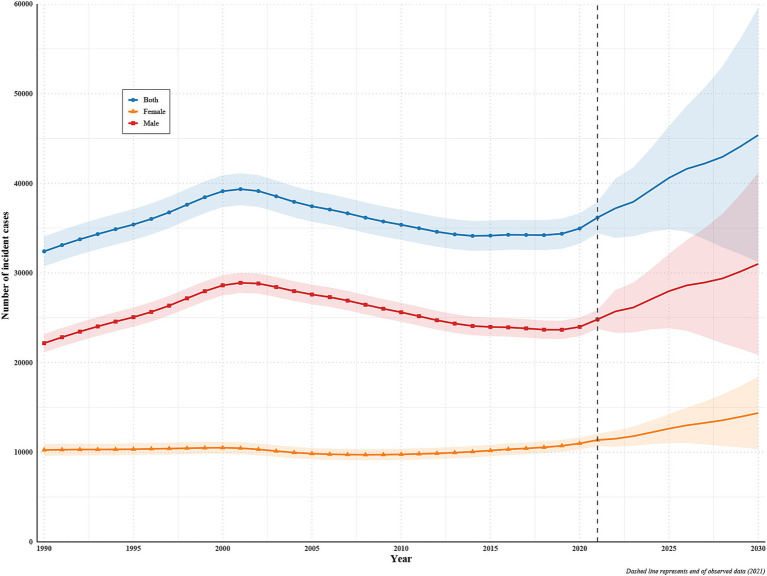
Projected trends in incident cases of disease among young people (2022–2030).

**Figure 4:**
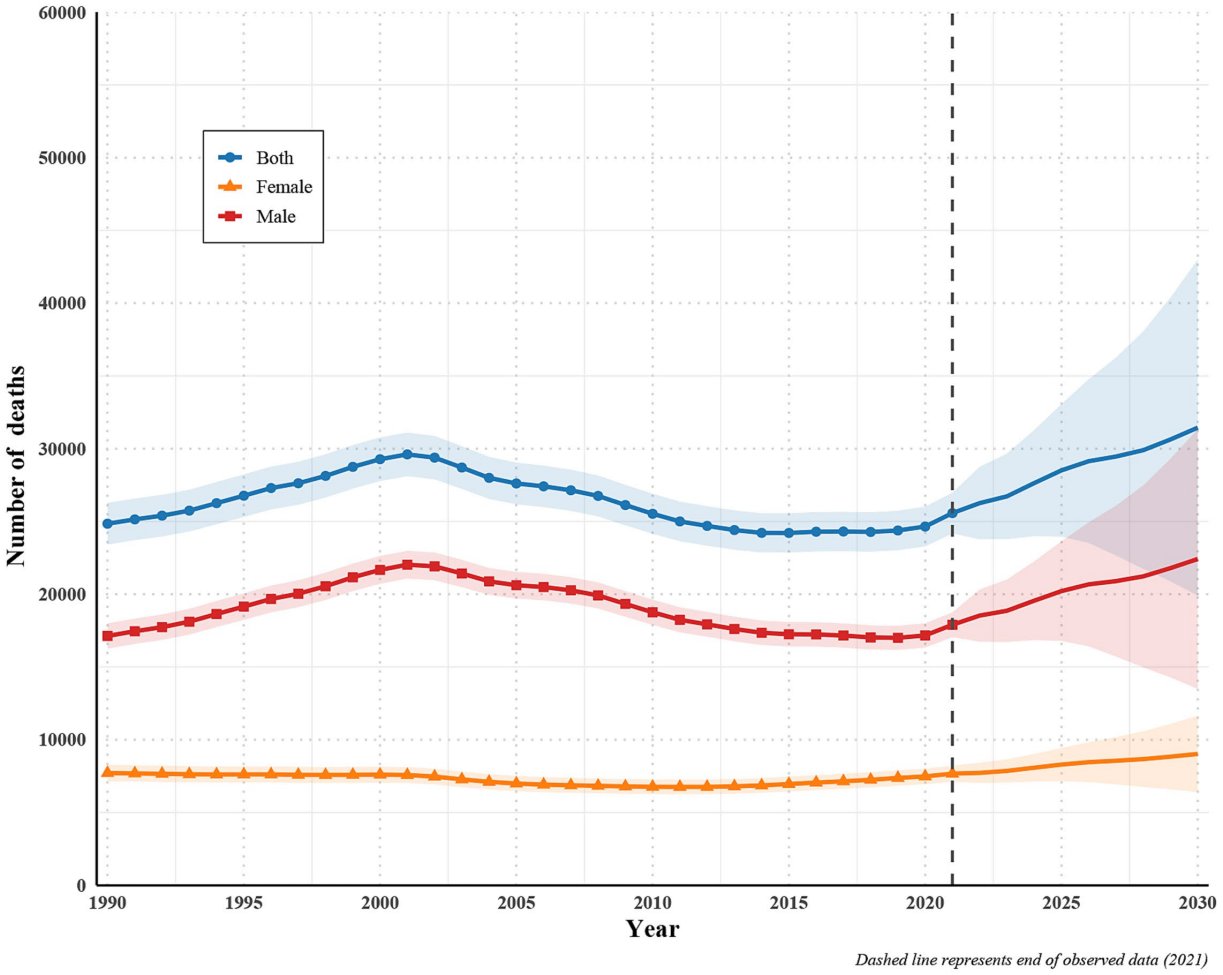
Projected trends in deaths cases of disease among young people (2022–2030).

Using the observed series (1990–2021) and a Bayesian age–period–cohort model, we project a continued rise in both incident and death counts through 2030, with persistently higher burdens in males than females (Figures 3, 4). For incidence, total cases are projected to increase from 36,180 in 2021 to 45,352 in 2030 (uncertainty interval [UI] 31,028–59,675), a + 25.3% change; male cases rise from 24,818 to 30,948 (UI 20,815–41,081; +24.7%), and female cases from 11,361 to 14,404 (UI 10,214–18,594; +26.8%). For deaths, totals increase from 25,578 in 2021 to 31,448 in 2030 (UI 19,909–42,987; +22.9%); male deaths rise from 17,903 to 22,422 (UI 13,498–31,347; +25.2%), while female deaths increase from 7,675 to 9,025 (UI 6,411–11,639; +17.6%) (Supplementary Table S11).

### Health inequality in liver cancer among

Based on the Slope Index of Inequality (SII) estimated using robust regression, all three indicators—incidence, deaths, and DALYs—demonstrated persistent but gradually narrowing health disparities in liver cancer among AYAs from 1990 to 2021 (Supplementary Figures S7–S9). The SII for incidence improved from −0.29 in 1990 to 0.01 in 2021, suggesting an almost complete attenuation of inequality in new liver cancer cases across countries with different SDI levels. For deaths, the SII increased from −0.38 to −0.20, indicating a moderate reduction in mortality inequality. Meanwhile, the SII for DALYs decreased substantially from −22.55 to −11.58, reflecting a marked convergence in overall disease burden.

## Discussion

This comprehensive analysis of the global burden of liver cancer among Adolescents and Young Adults (AYAs) from 1990 to 2030 reveals a complex and evolving epidemiological landscape. The central finding of our study is the dissociation between favorable trends in age-standardized rates and the rising absolute burden, projected to persist through 2030. This underscores a critical public health challenge: despite progress in population-level control, the growing and aging global population is leading to an increasing number of young individuals affected by liver cancer.

The observed decline in ASIR and ASMR since the early 2000s, particularly the sharp drop between 2001 and 2005, is an encouraging sign. This trend likely reflects the successful implementation of public health interventions over the past decades. The most significant factor is undoubtedly the widespread rollout of hepatitis B virus (HBV) vaccination programs, especially in endemic regions like East Asia (24, 25). Additionally, improved screening and control of other risk factors, such as hepatitis C virus (HCV) infection, along with advancements in managing chronic liver disease, have contributed to preventing cancer progression (24, 25).

However, these achievements represent only partial success, insufficient to curb the rising volume of disease in this young population. The discrepancy underscores missed opportunities in primary prevention. Despite WHO recommendations, global HBV birth-dose coverage remains suboptimal, especially in low-SDI regions (26). Our findings of persistently high incidence in Sub-Saharan Africa and South Asia—areas with known vaccination gaps—are consistent with these systemic failures (27). In contrast, the stabilization and mild resurgence in ASIR observed after 2015 warrant close attention. This may be driven by the growing impact of metabolic dysfunction-associated steatotic liver disease (MASLD) and rising obesity rates among younger populations, particularly in high-SDI countries, as well as incomplete control of viral hepatitis in other regions.

These findings reflect the success of national HBV immunization programs, particularly in countries like China, where birth-dose coverage exceeded 90% by 2015 (28). Additionally, improved access to nucleos(t)ide analogues (e.g., entecavir, tenofovir) for HBV suppression likely contributed to mortality reduction in this region (29). In contrast, South Asia experienced a rise in both absolute incidence and rate, indicating incomplete vaccination coverage and low awareness of HBV transmission pathways (30). These trends mirror gaps in perinatal HBV transmission control, where screening and antiviral prophylaxis for HBV-positive mothers remain rare (31). Studies have shown that combining vaccination with maternal tenofovir prophylaxis in late pregnancy significantly reduces vertical transmission, yet access remains highly unequal (32). Our results challenge this view and call for early-life intervention and risk-based surveillance in young populations. Males consistently showed higher incidence and mortality than females across all regions. This sex gap may be explained by biological factors such as androgen-driven carcinogenesis (33) and behavioral risks including alcohol use and delayed health engagement (34). Notably, while incidence rates between sexes are comparable in some high-SDI settings, the DALYs and mortality gap remains wide in low- and middle-SDI countries, indicating access inequities in care pathways. WHO recommends ultrasound plus AFP testing every 6 months for high-risk individuals (35), but implementation is hampered by lack of awareness, health infrastructure, and cost—especially in resource-limited settings (36). For AYAs with vertically acquired HBV, regular liver surveillance is often overlooked due to misperceived low risk and poor risk stratification tools (37). In many countries, diagnostic delays stem from limited access to imaging, specialist referral, and pathology services. Expanding point-of-care ultrasound, telemedicine-based triage, and integrated HBV-HIV platforms could enhance detection in under-resourced settings.

Our study confirms stark SDI-based disparities: high-SDI countries achieved significant reductions in mortality and DALYs, while low- and middle-SDI countries stagnated. In 1990, low-SDI regions showed the highest mortality rate, and in 2021, the burden had only shifted modestly toward low-middle SDI areas. These patterns highlight systemic challenges—underfunded health systems, fragmented HBV programs, lack of diagnostics, and weak civil registration systems. Addressing these will require investment not only in biomedical interventions but also in governance and financing reforms.

Our projections indicate that liver cancer cases among AYAs will rise from over 36 thousand in 2021 to approximately 45 thousand by 2030. This growth will be driven not by rising rates, but by inertia in prevention and expanding youth populations, especially in low-income countries. Without a rapid acceleration of HBV elimination programs and youth-focused screening, this growing burden will place increasing strain on already overstretched health systems.

To address these challenges, we propose a multi-pronged strategy: Strengthen HBV birth dose and infant immunization programs, especially in regions with low facility delivery rates. Implement maternal screening and tenofovir prophylaxis to prevent mother-to-child transmission. Expand youth-friendly CHB testing and care, including linkage to antivirals for eligible patients. Integrate liver cancer screening into HIV and reproductive health services, using risk-based models to identify candidates. Develop national liver cancer strategies that explicitly include adolescents and young adults—currently an overlooked group. Tackle structural inequalities by expanding financing, workforce capacity, and access to diagnostics in low-SDI settings.

## Conclusion

In conclusion, this study demonstrates a decline in age-standardized rates of liver cancer among AYAs since the early 2000s, yet projects a rising absolute burden through 2030. Significant sex-based and socioeconomic disparities persist. These findings underscore the critical need for enhanced, equitable prevention strategies—including bolstered HBV vaccination and targeted early detection—tailored to this young population to mitigate the growing disease burden.

## Data Availability

The original contributions presented in the study are included in the article/Supplementary material, further inquiries can be directed to the corresponding author/s.

## Footnotes

1
http://ghdx.healthdata.org
