# Supplementary material for: The descriptive epidemiology and projection of liver cancer in adolescents and young adults: findings from the global burden of disease study 2021
Source: Front Med (Lausanne). 2025 Dec 16;12:1690010. doi: 10.3389/fmed.2025.1690010 (PMC12750614; doi:10.3389/fmed.2025.1690010)

**Supplementary Figures**

Figure S1. The mean age-standardized incidence rate among individuals aged 15–39 years in 1990


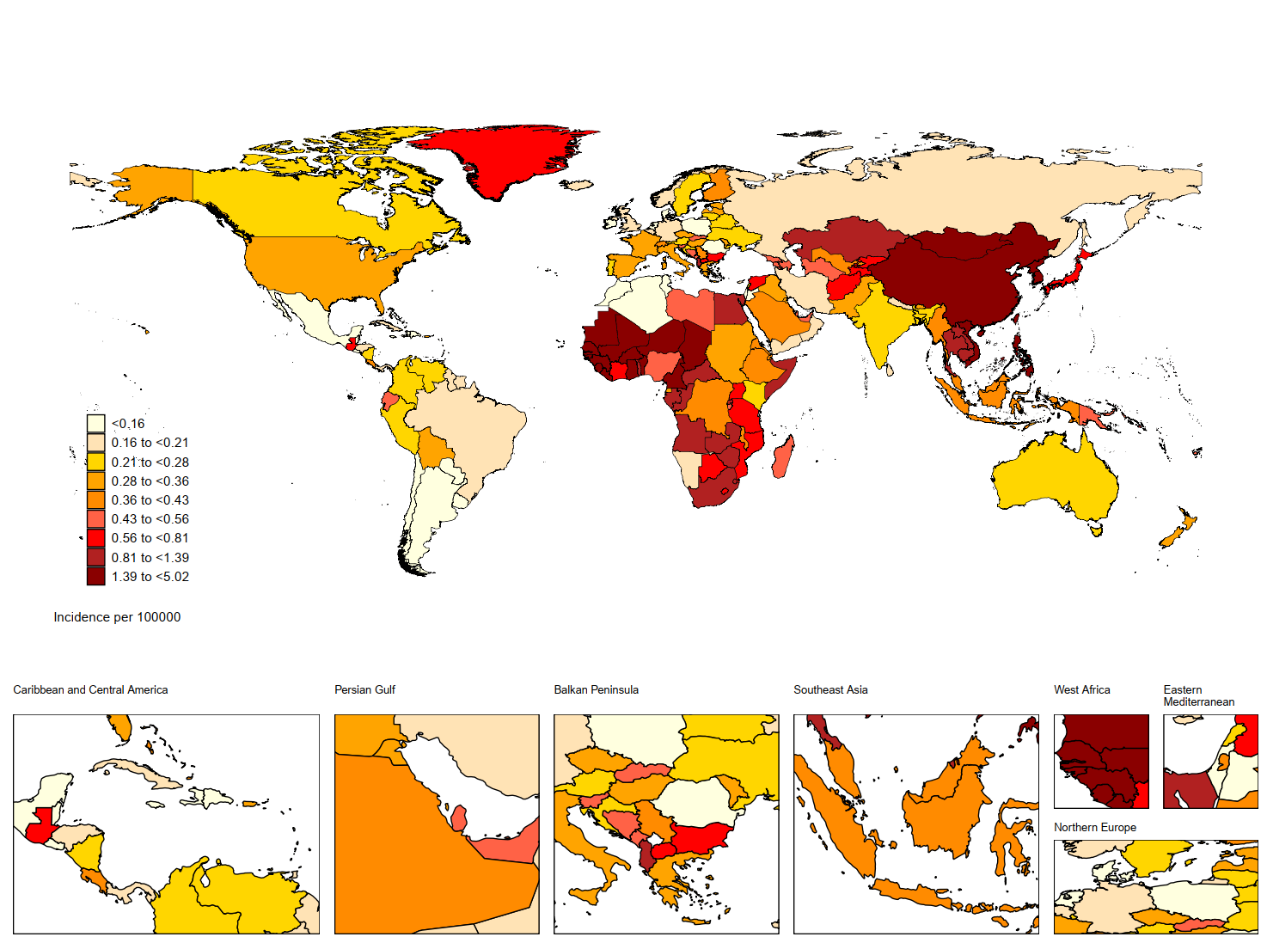


Figure S2. The mean age-standardized incidence rate among individuals aged 15–39 years in 2021


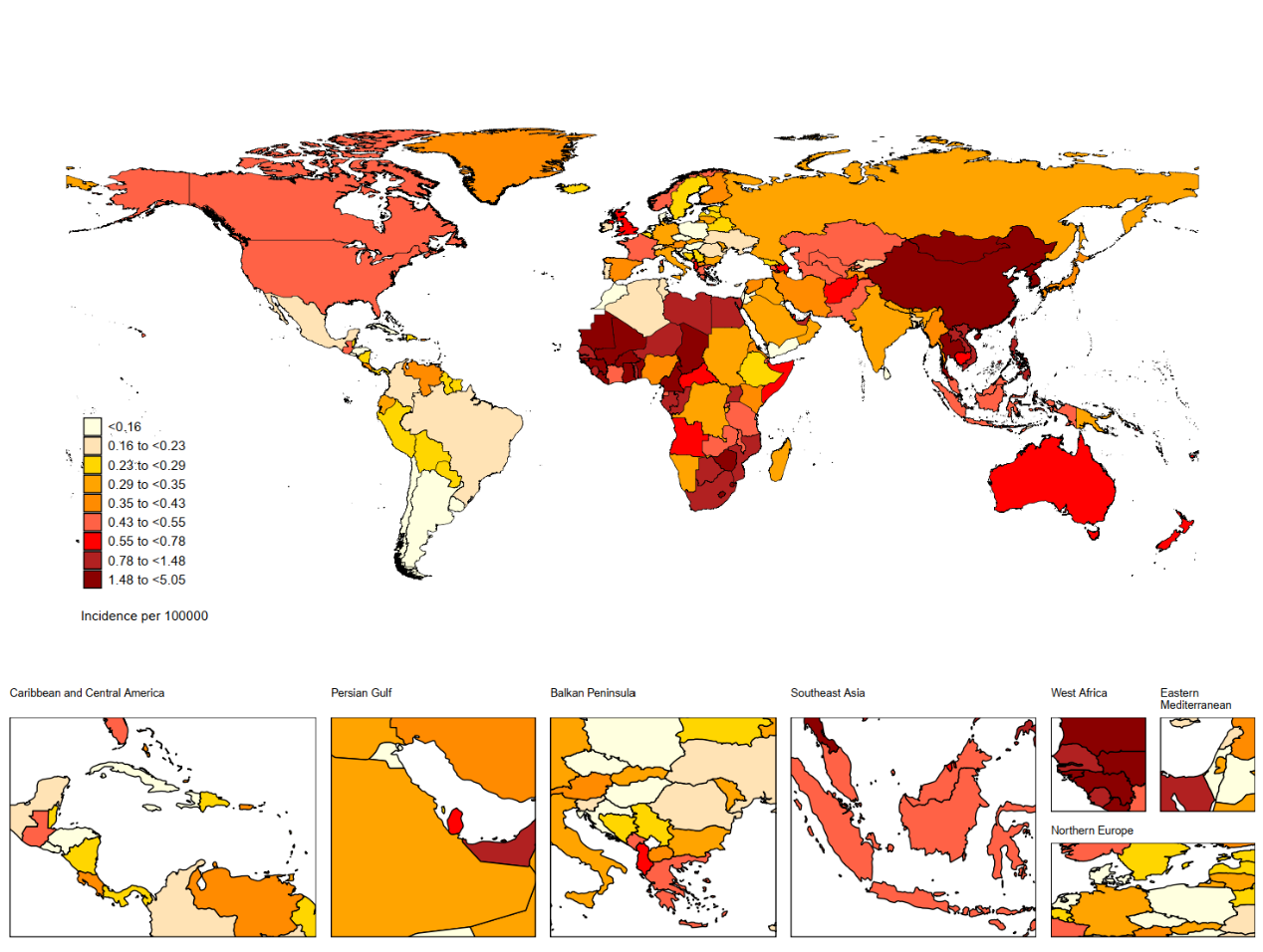


Figure S3. The global average mortality rate among individuals aged 15–39 years in 1990.


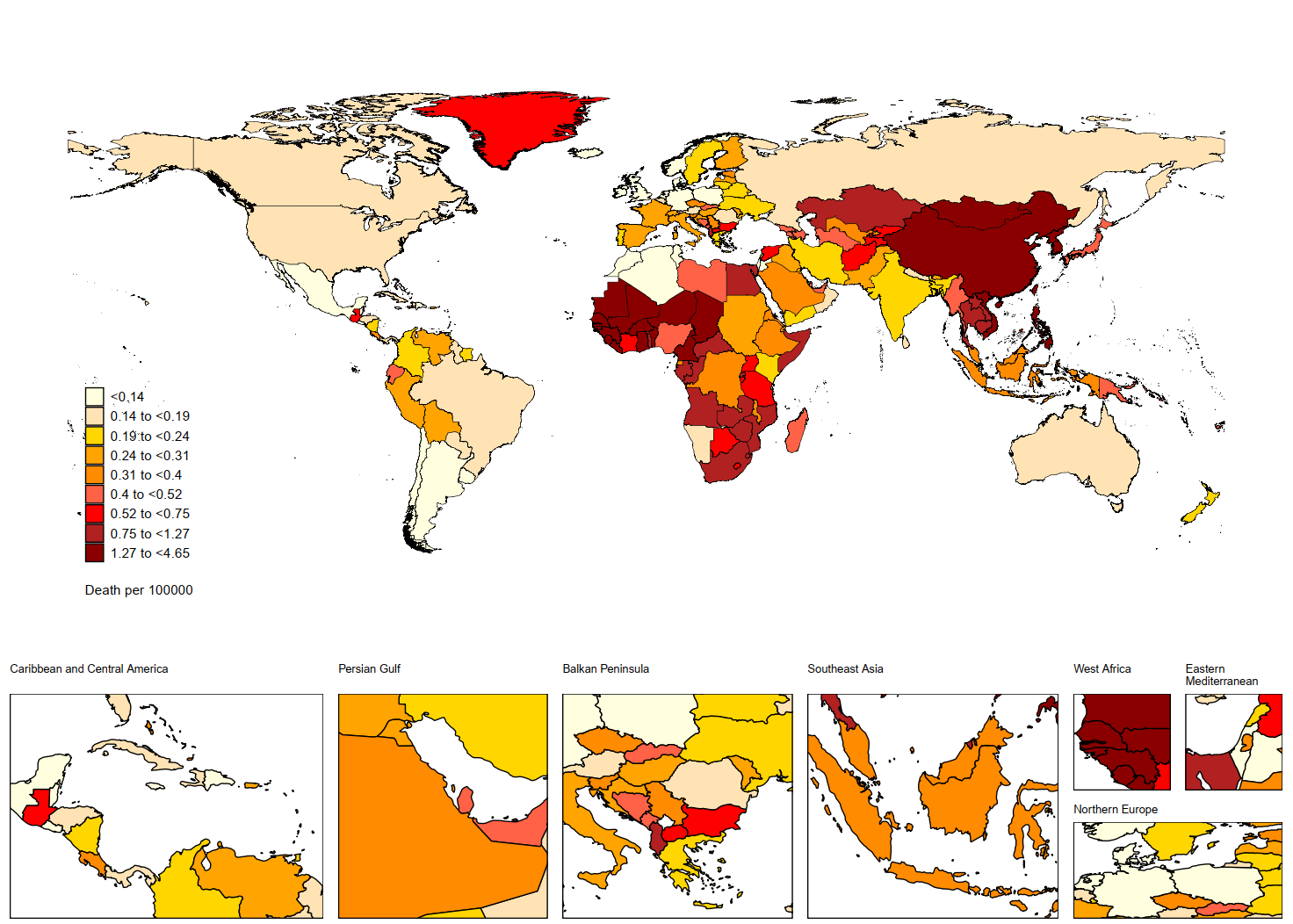


Figure S4. The global average mortality rate among individuals aged 15–39 years in 2021.


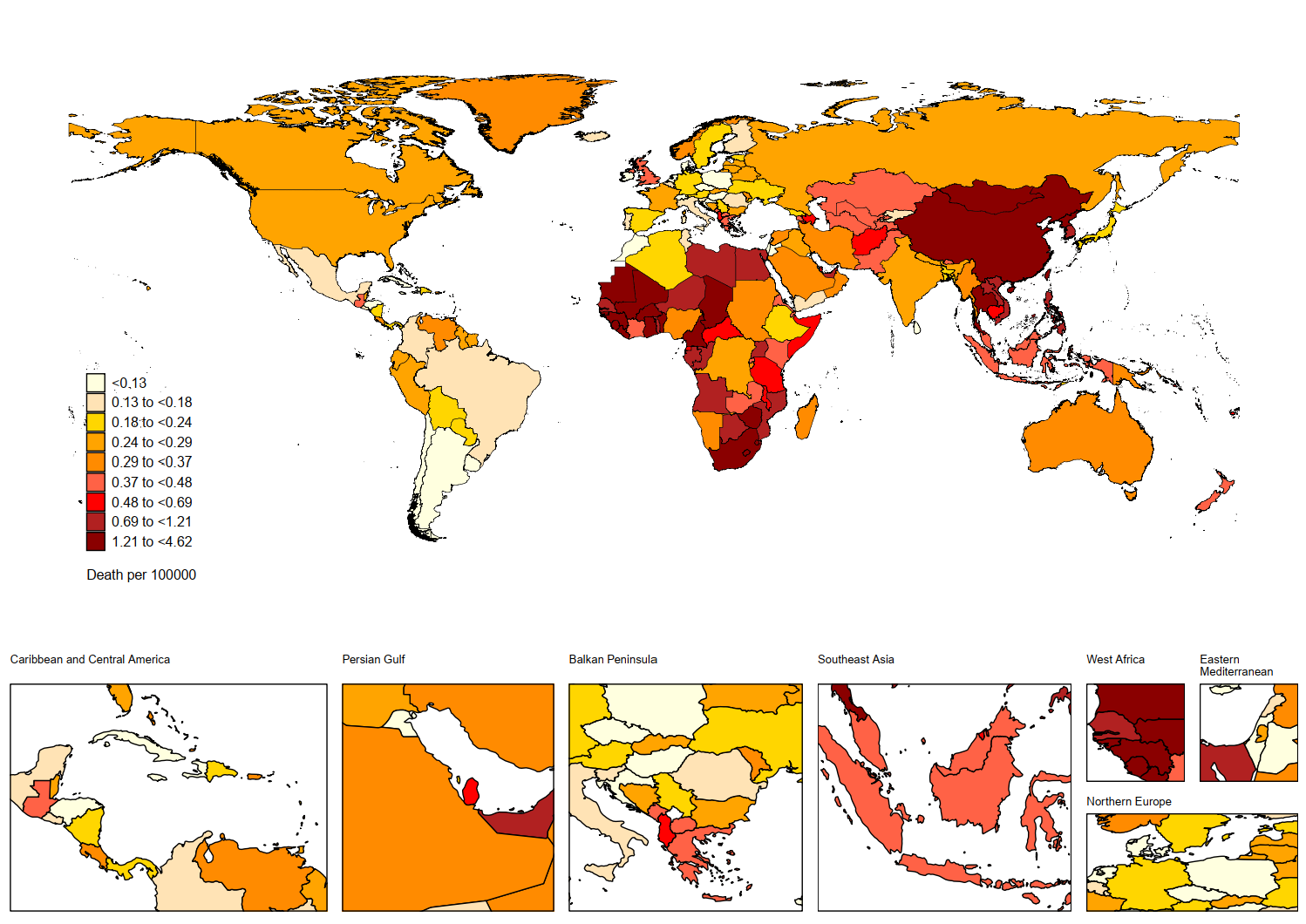


Figure S5. The disability-adjusted life years among individuals aged 15–39 years in 1990.


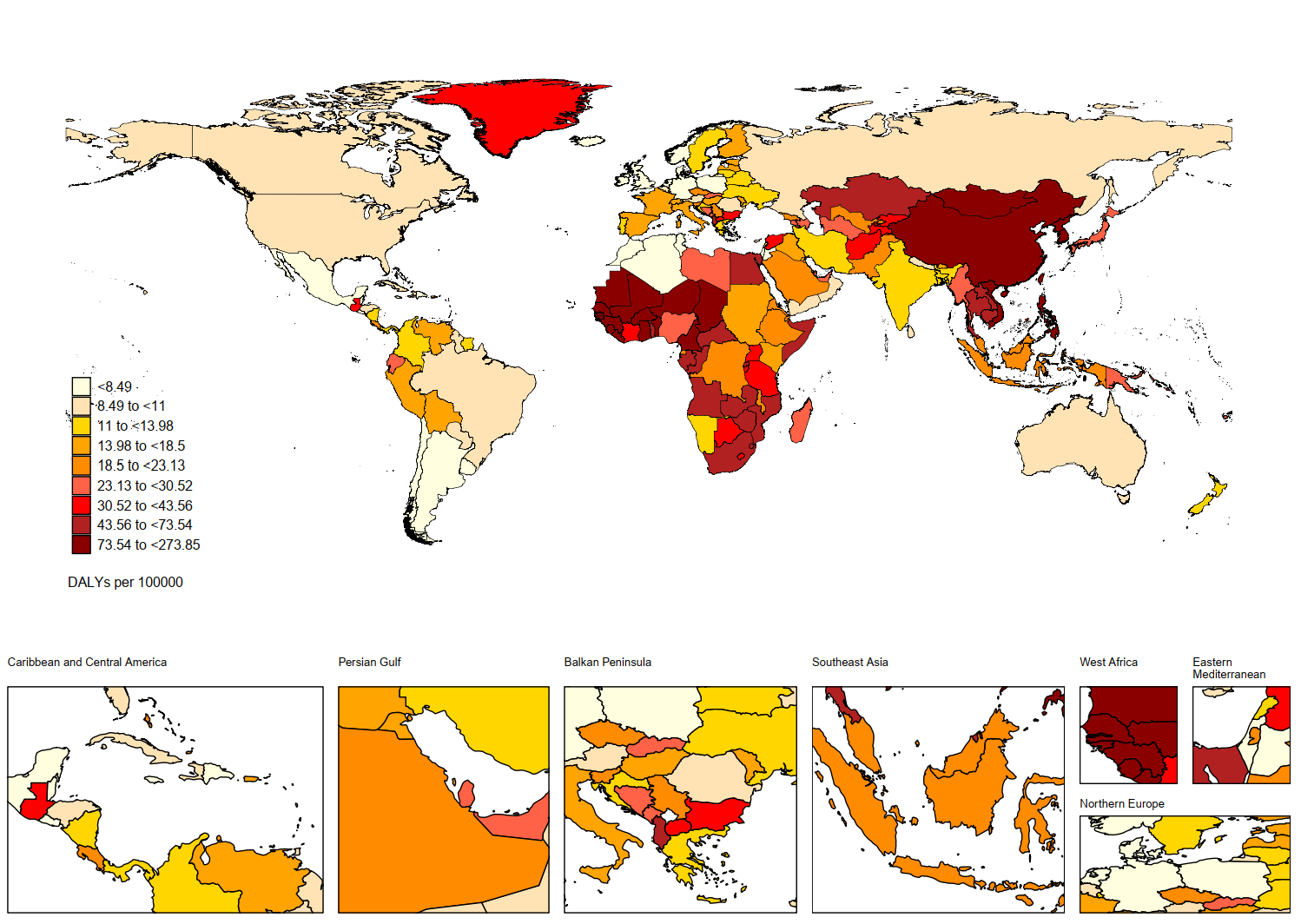


Figure S6. The disability-adjusted life years among individuals aged 15–39 years in


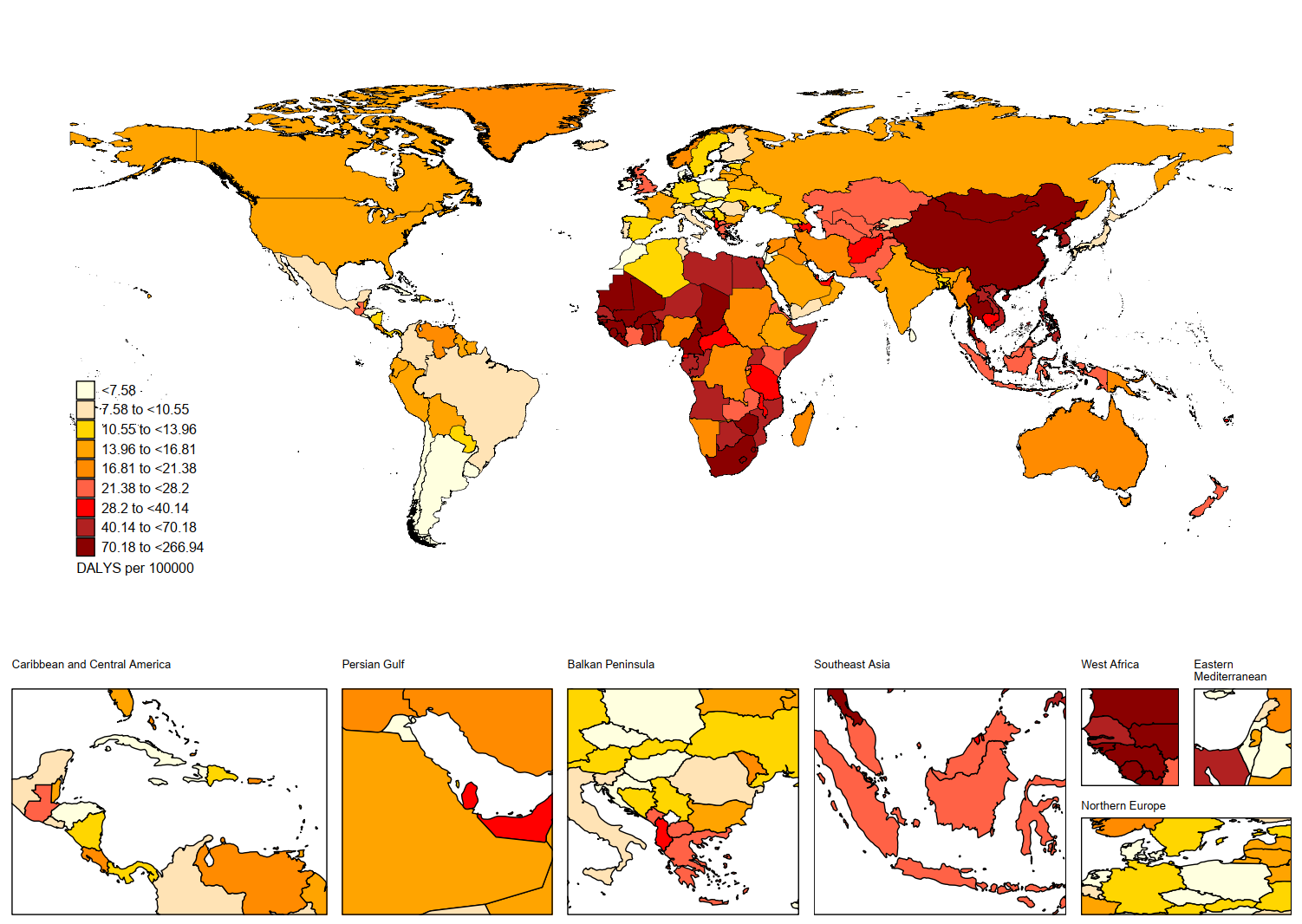
2021.


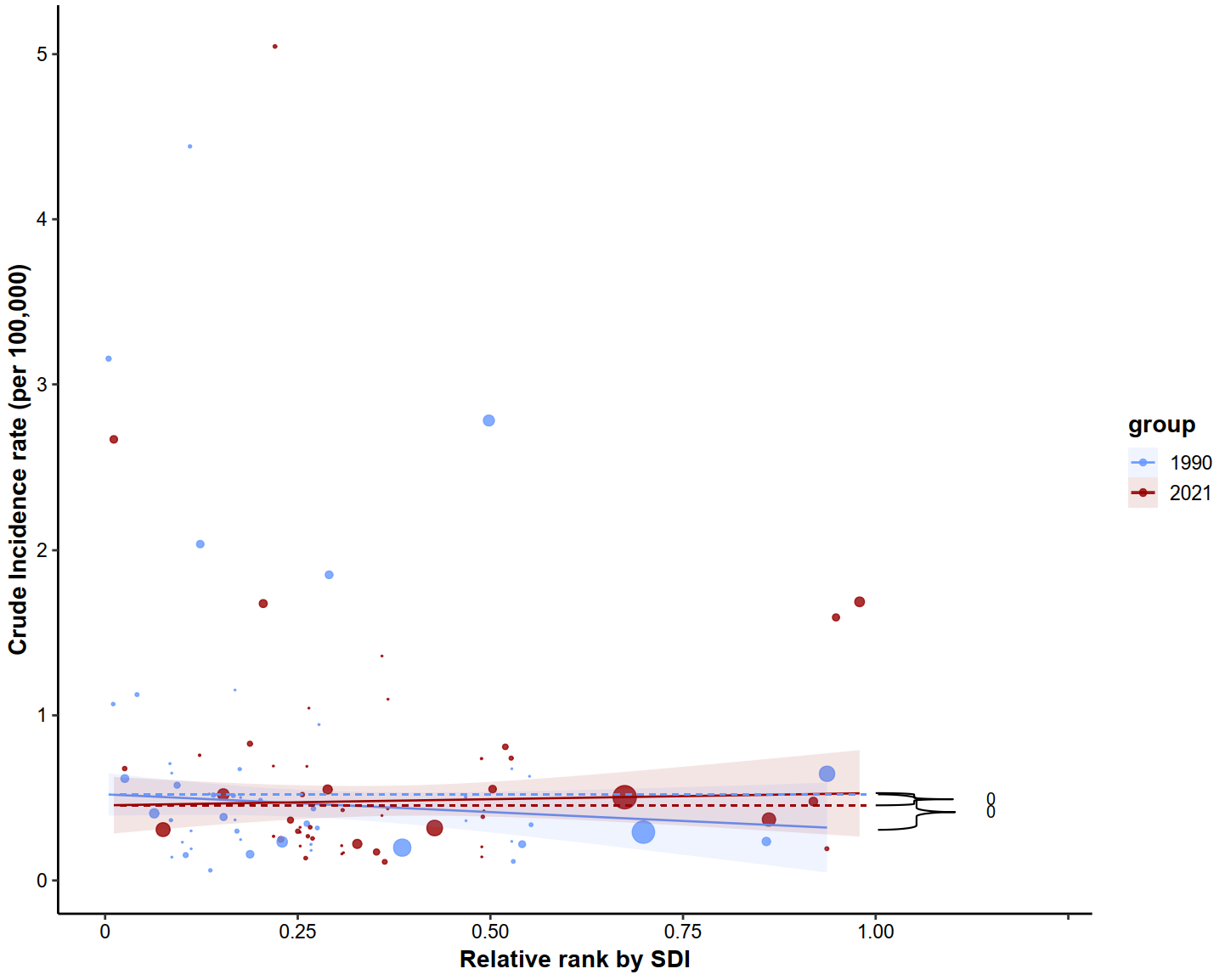
Figure S7. Slope Index of Inequality (SII) in Incidence of Liver Cancer among Adolescents and Young Adults (Aged 15–39 Years), 1990–2021

Figure S8. Slope Index of Inequality (SII) in Mortality from Liver Cancer among Adolescents and Young Adults (Aged 15–39 Years), 1990–2021


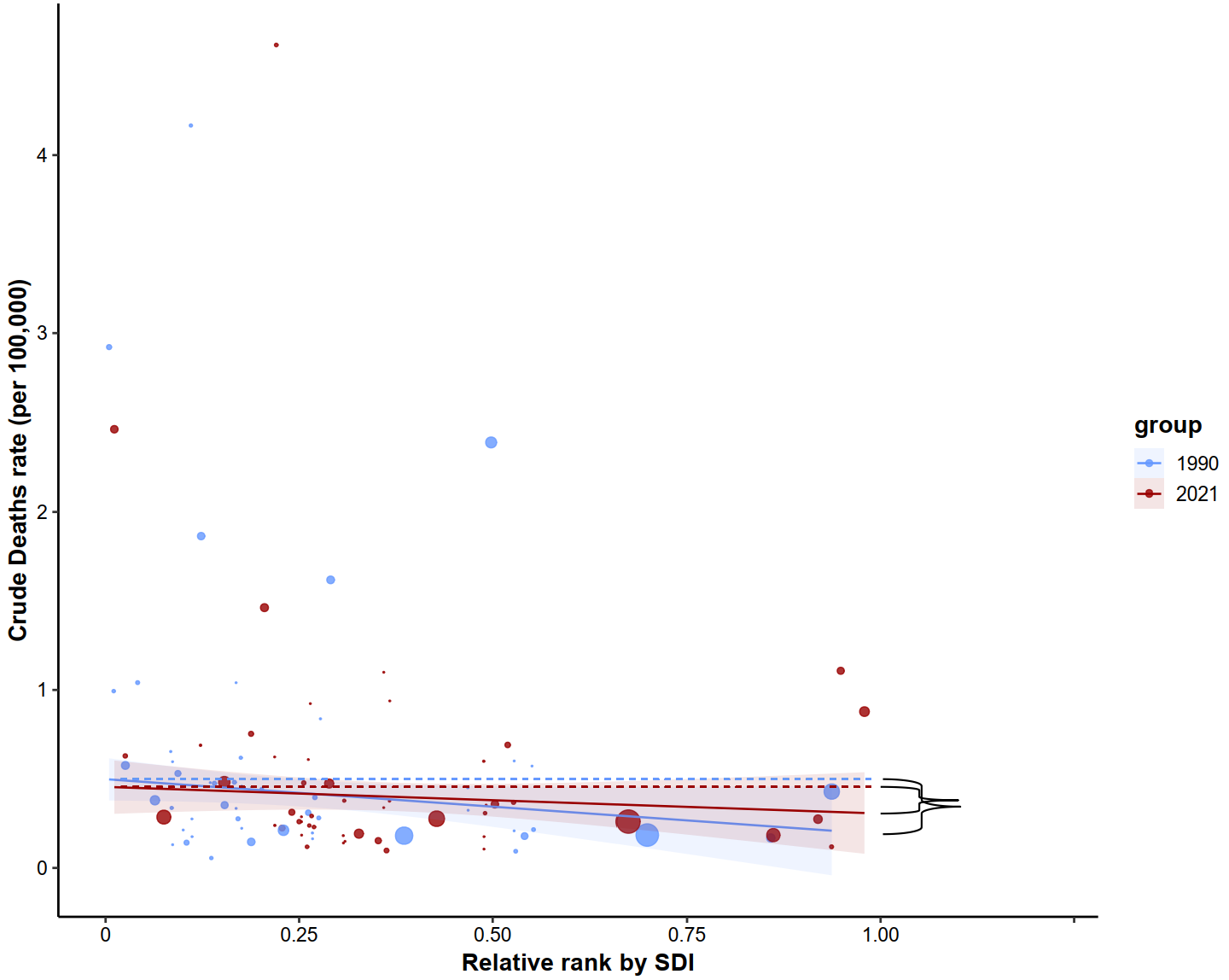


Figure S9. Slope Index of Inequality (SII) in DALYs due to Liver Cancer among Adolescents and Young Adults (Aged 15–39 Years), 1990–2021


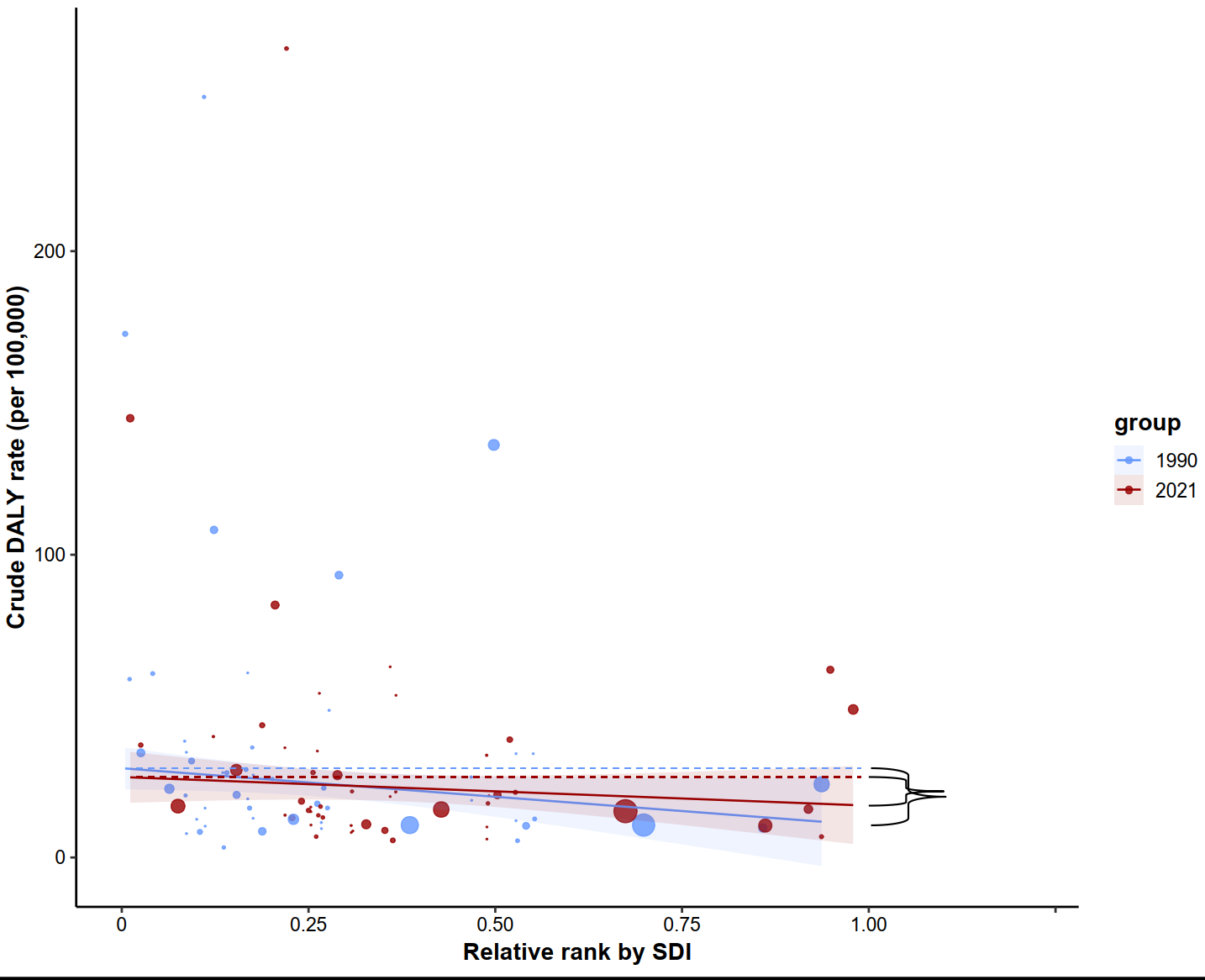

Supplement: Supplementary file 1 [file Data_Sheet_1.docx]
