# Supplementary material for: The descriptive epidemiology and projection of liver cancer in adolescents and young adults: findings from the global burden of disease study 2021
Source: Front Med (Lausanne). 2025 Dec 16;12:1690010. doi: 10.3389/fmed.2025.1690010 (PMC12750614; doi:10.3389/fmed.2025.1690010)
Supplement: Supplementary file 2 [file Table_1.docx]

| **Table S1. Age-standardized incidence rate of liver cancer in adolescents and youth (15–39 years) by country** | | | |
| --- | --- | --- | --- |
| **Location** | **Incidence in 1990(per 100,000)** | **Incidence in 2021(per 100,000)** |  |
| Algeria | 0.14 (0.11-0.19) | 0.22 (0.15-0.32) |  |
| Angola | 1.22 (0.17-3.88) | 0.75 (0.17-2.2) |  |
| Egypt | 0.87 (0.62-1.26) | 0.96 (0.73-1.26) |  |
| Ethiopia | 0.39 (0.25-0.58) | 0.25 (0.17-0.37) |  |
| Ghana | 2.59 (1.53-4.13) | 1.85 (1.14-3.02) |  |
| Kenya | 0.25 (0.18-0.37) | 0.41 (0.29-0.59) |  |
| Morocco | 0.04 (0.03-0.05) | 0.05 (0.03-0.08) |  |
| Nigeria | 0.5 (0.21-0.97) | 0.38 (0.21-0.63) |  |
| South Africa | 1.27 (0.82-1.9) | 1.39 (1.14-1.67) |  |
| Uganda | 0.65 (0.43-0.99) | 0.84 (0.56-1.19) |  |
| Argentina | 0.05 (0.04-0.06) | 0.1 (0.09-0.12) |  |
| Brazil | 0.17 (0.17-0.18) | 0.17 (0.16-0.18) |  |
| Canada | 0.24 (0.21-0.27) | 0.48 (0.4-0.58) |  |
| Chile | 0.07 (0.06-0.09) | 0.14 (0.11-0.16) |  |
| Colombia | 0.24 (0.22-0.26) | 0.18 (0.15-0.22) |  |
| Mexico | 0.13 (0.13-0.13) | 0.19 (0.17-0.21) |  |
| Peru | 0.26 (0.18-0.37) | 0.28 (0.19-0.41) |  |
| United States of America | 0.29 (0.28-0.3) | 0.5 (0.48-0.53) |  |
| Bangladesh | 0.21 (0.15-0.31) | 0.23 (0.14-0.39) |  |
| Cambodia | 0.88 (0.47-1.57) | 0.71 (0.34-1.41) |  |
| China | 2.22 (1.84-2.66) | 2.57 (2.02-3.3) |  |
| Democratic People's Republic of Korea | 2.04 (0.98-3.65) | 1.68 (0.88-3.08) |  |
| India | 0.22 (0.19-0.26) | 0.3 (0.25-0.36) |  |
| Indonesia | 0.4 (0.26-0.59) | 0.48 (0.3-0.76) |  |
| Japan | 0.65 (0.62-0.68) | 0.37 (0.33-0.41) |  |
| Malaysia | 0.38 (0.29-0.52) | 0.55 (0.4-0.78) |  |
| Myanmar | 0.43 (0.18-0.84) | 0.39 (0.19-0.78) |  |
| Nepal | 0.18 (0.12-0.27) | 0.29 (0.19-0.42) |  |
| Pakistan | 0.33 (0.25-0.44) | 0.48 (0.36-0.65) |  |
| Philippines | 1.42 (1.03-1.66) | 0.88 (0.72-1.05) |  |
| Republic of Korea | 2.78 (1.89-3.94) | 1.69 (1.24-2.28) |  |
| Singapore | 0.73 (0.63-0.85) | 0.41 (0.33-0.51) |  |
| Sri Lanka | 0.2 (0.15-0.27) | 0.15 (0.1-0.23) |  |
| Taiwan (Province of China) | 1.85 (1.66-2.04) | 1.59 (1.31-1.94) |  |
| Thailand | 1.37 (0.94-1.96) | 2.07 (1.36-3.0) |  |
| Viet Nam | 1.46 (1.03-2.04) | 1.39 (0.91-2.24) |  |
| Austria | 0.23 (0.2-0.26) | 0.4 (0.33-0.46) |  |
| Belgium | 0.18 (0.16-0.21) | 0.27 (0.23-0.32) |  |
| Czechia | 0.36 (0.31-0.42) | 0.13 (0.1-0.16) |  |
| Denmark | 0.16 (0.14-0.19) | 0.11 (0.09-0.13) |  |
| Finland | 0.38 (0.32-0.46) | 0.36 (0.29-0.44) |  |
| France | 0.32 (0.28-0.35) | 0.44 (0.38-0.53) |  |
| Germany | 0.17 (0.14-0.19) | 0.31 (0.27-0.37) |  |
| Italy | 0.35 (0.32-0.38) | 0.34 (0.3-0.38) |  |
| Netherlands | 0.1 (0.09-0.12) | 0.14 (0.11-0.17) |  |
| Norway | 0.18 (0.17-0.19) | 0.49 (0.43-0.56) |  |
| Poland | 0.06 (0.05-0.06) | 0.13 (0.12-0.15) |  |
| Spain | 0.34 (0.3-0.38) | 0.42 (0.34-0.51) |  |
| Sweden | 0.23 (0.21-0.26) | 0.27 (0.24-0.32) |  |
| United Kingdom | 0.19 (0.18-0.19) | 0.74 (0.71-0.78) |  |
| Australia | 0.22 (0.19-0.25) | 0.55 (0.47-0.65) |  |
| Fiji | 0.53 (0.36-0.78) | 0.64 (0.41-0.96) |  |
| New Zealand | 0.34 (0.29-0.4) | 0.74 (0.61-0.89) |  |
| Papua New Guinea | 0.44 (0.17-1.08) | 0.34 (0.16-0.77) |  |
| Afghanistan | 0.67 (0.47-0.96) | 0.6 (0.42-0.88) |  |
| Albania | 0.89 (0.66-1.16) | 0.58 (0.42-0.8) |  |
| American Samoa | 0.5 (0.35-0.72) | 1.04 (0.68-1.5) |  |
| Andorra | 0.87 (0.58-1.28) | 0.99 (0.6-1.47) |  |
| Antigua and Barbuda | 0.25 (0.22-0.28) | 0.16 (0.14-0.19) |  |
| Armenia | 0.49 (0.41-0.6) | 0.43 (0.36-0.52) |  |
| Azerbaijan | 0.54 (0.31-0.87) | 0.6 (0.33-1.03) |  |
| Bahamas | 0.36 (0.32-0.42) | 0.4 (0.32-0.5) |  |
| Bahrain | 0.33 (0.25-0.46) | 0.25 (0.17-0.35) |  |
| Barbados | 0.22 (0.2-0.25) | 0.21 (0.17-0.28) |  |
| Belarus | 0.22 (0.18-0.27) | 0.28 (0.21-0.35) |  |
| Belize | 0.14 (0.12-0.17) | 0.27 (0.23-0.3) |  |
| Benin | 2.65 (1.35-4.2) | 1.83 (1.17-2.61) |  |
| Bermuda | 0.24 (0.21-0.26) | 0.14 (0.11-0.18) |  |
| Bhutan | 0.31 (0.17-0.5) | 0.41 (0.24-0.67) |  |
| Bolivia (Plurinational State of) | 0.32 (0.21-0.45) | 0.26 (0.17-0.42) |  |
| Bosnia and Herzegovina | 0.52 (0.43-0.62) | 0.27 (0.19-0.35) |  |
| Botswana | 0.72 (0.3-1.53) | 1.05 (0.44-2.49) |  |
| Brunei Darussalam | 0.94 (0.68-1.34) | 0.74 (0.54-1.02) |  |
| Bulgaria | 0.64 (0.49-0.8) | 0.29 (0.21-0.4) |  |
| Burkina Faso | 3.16 (1.32-7.15) | 2.67 (1.15-5.18) |  |
| Burundi | 0.55 (0.34-1.02) | 0.33 (0.2-0.54) |  |
| Cabo Verde | 2.16 (1.51-3.04) | 2.27 (1.57-3.22) |  |
| Cameroon | 2.54 (1.75-3.86) | 1.98 (1.1-3.47) |  |
| Central African Republic | 1.07 (0.39-2.27) | 0.68 (0.26-1.51) |  |
| Chad | 2.06 (0.81-4.52) | 1.69 (0.9-3.2) |  |
| Comoros | 0.56 (0.25-0.85) | 0.56 (0.38-0.85) |  |
| Congo | 1.16 (0.49-2.31) | 0.78 (0.33-1.63) |  |
| Cook Islands | 1.15 (0.79-1.63) | 1.36 (0.88-2.0) |  |
| Costa Rica | 0.37 (0.32-0.41) | 0.42 (0.35-0.51) |  |
| Croatia | 0.28 (0.21-0.35) | 0.14 (0.11-0.19) |  |
| Cuba | 0.2 (0.17-0.24) | 0.13 (0.1-0.16) |  |
| Cyprus | 0.17 (0.12-0.25) | 0.19 (0.14-0.27) |  |
| Cote d'Ivoire | 0.73 (0.43-1.15) | 0.48 (0.28-0.74) |  |
| Democratic Republic of the Congo | 0.41 (0.2-0.87) | 0.31 (0.14-0.71) |  |
| Djibouti | 0.37 (0.19-0.65) | 0.48 (0.25-0.83) |  |
| Dominica | 0.16 (0.1-0.28) | 0.24 (0.16-0.34) |  |
| Dominican Republic | 0.15 (0.11-0.21) | 0.25 (0.17-0.34) |  |
| Ecuador | 0.53 (0.46-0.6) | 0.31 (0.24-0.39) |  |
| El Salvador | 0.14 (0.12-0.16) | 0.15 (0.12-0.19) |  |
| Equatorial Guinea | 0.36 (0.18-0.78) | 0.53 (0.25-0.96) |  |
| Eritrea | 0.39 (0.23-0.65) | 0.42 (0.24-0.75) |  |
| Estonia | 0.36 (0.3-0.42) | 0.26 (0.21-0.31) |  |
| Eswatini | 1.06 (0.4-2.07) | 3.79 (1.12-9.64) |  |
| Gabon | 0.98 (0.39-2.25) | 0.91 (0.48-1.61) |  |
| Gambia | 3.52 (2.32-5.05) | 4.54 (2.77-7.38) |  |
| Georgia | 0.44 (0.37-0.51) | 0.25 (0.22-0.3) |  |
| Greece | 0.28 (0.26-0.3) | 0.55 (0.49-0.62) |  |
| Greenland | 0.63 (0.45-0.87) | 0.42 (0.26-0.63) |  |
| Grenada | 0.23 (0.18-0.3) | 0.29 (0.24-0.36) |  |
| Guam | 0.36 (0.3-0.45) | 1.1 (0.84-1.38) |  |
| Guatemala | 0.57 (0.53-0.61) | 0.44 (0.39-0.51) |  |
| Guinea | 3.31 (2.36-4.61) | 2.67 (1.69-4.0) |  |
| Guinea-Bissau | 4.68 (1.92-7.49) | 3.27 (1.93-4.93) |  |
| Guyana | 0.19 (0.16-0.24) | 0.27 (0.2-0.34) |  |
| Haiti | 0.16 (0.09-0.27) | 0.13 (0.06-0.24) |  |
| Honduras | 0.17 (0.12-0.23) | 0.14 (0.08-0.22) |  |
| Hungary | 0.34 (0.27-0.44) | 0.11 (0.09-0.15) |  |
| Iceland | 0.17 (0.15-0.19) | 0.27 (0.22-0.33) |  |
| Iran (Islamic Republic of) | 0.21 (0.18-0.26) | 0.36 (0.32-0.41) |  |
| Iraq | 0.33 (0.24-0.46) | 0.33 (0.23-0.5) |  |
| Ireland | 0.12 (0.1-0.13) | 0.19 (0.16-0.23) |  |
| Israel | 0.1 (0.08-0.11) | 0.14 (0.12-0.17) |  |
| Jamaica | 0.06 (0.05-0.08) | 0.14 (0.1-0.19) |  |
| Jordan | 0.12 (0.08-0.18) | 0.1 (0.07-0.14) |  |
| Kazakhstan | 0.96 (0.86-1.08) | 0.44 (0.36-0.53) |  |
| Kiribati | 1.14 (0.83-1.61) | 1.2 (0.77-1.8) |  |
| Kuwait | 0.33 (0.28-0.39) | 0.08 (0.06-0.1) |  |
| Kyrgyzstan | 0.68 (0.51-0.9) | 0.18 (0.12-0.25) |  |
| Lao People's Democratic Republic | 1.13 (0.72-1.61) | 0.83 (0.53-1.27) |  |
| Latvia | 0.3 (0.25-0.36) | 0.28 (0.22-0.36) |  |
| Lebanon | 0.25 (0.18-0.35) | 0.2 (0.15-0.26) |  |
| Lesotho | 0.71 (0.23-1.84) | 2.71 (0.89-7.87) |  |
| Liberia | 2.6 (1.25-4.25) | 2.65 (1.5-4.08) |  |
| Libya | 0.49 (0.34-0.68) | 0.85 (0.57-1.22) |  |
| Lithuania | 0.25 (0.22-0.29) | 0.3 (0.24-0.39) |  |
| Luxembourg | 0.24 (0.22-0.25) | 0.24 (0.21-0.27) |  |
| Madagascar | 0.48 (0.33-0.68) | 0.35 (0.23-0.55) |  |
| Malawi | 0.39 (0.25-0.58) | 0.52 (0.34-0.78) |  |
| Maldives | 0.41 (0.22-0.66) | 0.42 (0.26-0.65) |  |
| Mali | 2.84 (2.17-3.65) | 2.81 (1.96-3.84) |  |
| Malta | 0.1 (0.09-0.12) | 0.21 (0.18-0.26) |  |
| Marshall Islands | 0.43 (0.27-0.66) | 0.68 (0.4-1.18) |  |
| Mauritania | 5.02 (1.03-10.51) | 2.46 (1.2-4.12) |  |
| Mauritius | 0.34 (0.31-0.37) | 0.06 (0.06-0.07) |  |
| Micronesia (Federated States of) | 0.74 (0.47-1.12) | 0.77 (0.44-1.35) |  |
| Monaco | 0.36 (0.25-0.53) | 0.87 (0.51-1.38) |  |
| Mongolia | 4.44 (2.89-6.59) | 5.05 (3.47-7.28) |  |
| Montenegro | 0.5 (0.4-0.63) | 0.43 (0.33-0.57) |  |
| Mozambique | 0.81 (0.44-1.29) | 1.13 (0.52-2.36) |  |
| Namibia | 0.2 (0.11-0.37) | 0.35 (0.21-0.57) |  |
| Nauru | 1.2 (0.79-1.75) | 1.12 (0.59-1.84) |  |
| Nicaragua | 0.21 (0.17-0.27) | 0.24 (0.18-0.31) |  |
| Niger | 2.25 (0.99-4.48) | 1.07 (0.64-1.91) |  |
| Niue | 0.57 (0.33-0.96) | 0.65 (0.41-1.12) |  |
| North Macedonia | 0.67 (0.57-0.82) | 0.43 (0.32-0.54) |  |
| Northern Mariana Islands | 0.68 (0.42-1.02) | 0.39 (0.28-0.54) |  |
| Oman | 0.21 (0.12-0.35) | 0.35 (0.23-0.5) |  |
| Palau | 1.07 (0.56-1.91) | 1.98 (1.22-3.12) |  |
| Palestine | 0.37 (0.25-0.52) | 0.3 (0.23-0.38) |  |
| Panama | 0.2 (0.19-0.21) | 0.24 (0.2-0.29) |  |
| Paraguay | 0.16 (0.13-0.2) | 0.25 (0.17-0.36) |  |
| Portugal | 0.21 (0.18-0.25) | 0.23 (0.2-0.29) |  |
| Puerto Rico | 0.32 (0.26-0.39) | 0.39 (0.31-0.48) |  |
| Qatar | 0.51 (0.35-0.79) | 0.66 (0.46-0.91) |  |
| Republic of Moldova | 0.3 (0.28-0.32) | 0.32 (0.28-0.36) |  |
| Romania | 0.16 (0.13-0.2) | 0.17 (0.14-0.22) |  |
| Russian Federation | 0.2 (0.19-0.2) | 0.32 (0.3-0.34) |  |
| Rwanda | 0.81 (0.53-1.17) | 0.48 (0.3-0.76) |  |
| Saint Kitts and Nevis | 0.37 (0.32-0.42) | 0.17 (0.11-0.24) |  |
| Saint Lucia | 0.19 (0.16-0.22) | 0.21 (0.17-0.25) |  |
| Saint Vincent and the Grenadines | 0.3 (0.26-0.35) | 0.32 (0.28-0.37) |  |
| Samoa | 0.39 (0.24-0.59) | 0.45 (0.24-0.74) |  |
| San Marino | 0.12 (0.09-0.16) | 0.22 (0.13-0.34) |  |
| Sao Tome and Principe | 0.48 (0.3-0.69) | 0.45 (0.23-0.91) |  |
| Saudi Arabia | 0.39 (0.25-0.62) | 0.35 (0.22-0.5) |  |
| Senegal | 1.88 (1.05-2.84) | 1.28 (0.81-1.89) |  |
| Serbia | 0.39 (0.28-0.53) | 0.28 (0.19-0.41) |  |
| Seychelles | 0.8 (0.56-1.08) | 0.41 (0.28-0.6) |  |
| Sierra Leone | 2.23 (0.7-4.27) | 1.39 (0.88-2.12) |  |
| Slovakia | 0.45 (0.32-0.64) | 0.29 (0.18-0.44) |  |
| Slovenia | 0.44 (0.39-0.51) | 0.2 (0.16-0.24) |  |
| Solomon Islands | 0.71 (0.21-1.5) | 0.76 (0.45-1.19) |  |
| Somalia | 0.81 (0.34-1.49) | 0.72 (0.32-1.45) |  |
| South Sudan | 0.48 (0.29-0.76) | 0.62 (0.38-0.96) |  |
| Sudan | 0.32 (0.18-0.56) | 0.32 (0.19-0.51) |  |
| Suriname | 0.21 (0.13-0.29) | 0.28 (0.19-0.41) |  |
| Switzerland | 0.39 (0.32-0.47) | 0.21 (0.17-0.26) |  |
| Syrian Arab Republic | 0.58 (0.44-0.76) | 0.37 (0.25-0.52) |  |
| Tajikistan | 0.61 (0.36-0.96) | 0.43 (0.27-0.67) |  |
| Timor-Leste | 0.33 (0.18-0.57) | 0.23 (0.13-0.44) |  |
| Togo | 1.23 (0.84-1.87) | 1.15 (0.7-1.99) |  |
| Tokelau | 0.52 (0.29-0.97) | 0.69 (0.45-1.09) |  |
| Tonga | 1.91 (1.24-2.97) | 2.51 (1.5-4.24) |  |
| Trinidad and Tobago | 0.21 (0.19-0.23) | 0.3 (0.23-0.38) |  |
| Tunisia | 0.13 (0.09-0.18) | 0.22 (0.14-0.31) |  |
| Turkmenistan | 0.51 (0.46-0.57) | 0.52 (0.39-0.69) |  |
| Tuvalu | 0.65 (0.44-1.0) | 0.69 (0.44-1.07) |  |
| Turkey | 0.2 (0.15-0.26) | 0.2 (0.15-0.27) |  |
| Ukraine | 0.23 (0.19-0.28) | 0.22 (0.15-0.29) |  |
| United Arab Emirates | 0.49 (0.3-0.77) | 0.81 (0.51-1.18) |  |
| United Republic of Tanzania | 0.62 (0.42-0.85) | 0.52 (0.33-0.79) |  |
| United States Virgin Islands | 0.18 (0.12-0.27) | 0.2 (0.13-0.32) |  |
| Uruguay | 0.06 (0.05-0.07) | 0.15 (0.13-0.18) |  |
| Uzbekistan | 0.37 (0.27-0.48) | 0.48 (0.36-0.62) |  |
| Vanuatu | 0.56 (0.3-1.05) | 0.58 (0.34-0.97) |  |
| Venezuela (Bolivarian Republic of) | 0.26 (0.25-0.28) | 0.36 (0.28-0.46) |  |
| Yemen | 0.2 (0.07-0.46) | 0.15 (0.07-0.33) |  |
| Zambia | 1.05 (0.68-1.66) | 0.5 (0.18-1.4) |  |
| Zimbabwe | 1.09 (0.62-1.72) | 2.05 (1.2-3.2) |  |
