# Supplementary material for: The descriptive epidemiology and projection of liver cancer in adolescents and young adults: findings from the global burden of disease study 2021
Source: Front Med (Lausanne). 2025 Dec 16;12:1690010. doi: 10.3389/fmed.2025.1690010 (PMC12750614; doi:10.3389/fmed.2025.1690010)
Supplement: Supplementary file 3 [file Table_2.docx]

| **Table S2. Age-standardized mortality rate of liver cancer in youth (15–39 years) by country** | | |  |
| --- | --- | --- | --- |
| **Location** | **Mortality in 1990(per 100,000)** | **Mortality in 2021(per 100,000)** | |
| Afghanistan | 0.63 (0.44-0.9) | 0.56 (0.4-0.81) | |
| Albania | 0.83 (0.61-1.08) | 0.52 (0.37-0.72) | |
| Algeria | 0.13 (0.1-0.18) | 0.19 (0.13-0.27) | |
| American Samoa | 0.46 (0.32-0.65) | 0.92 (0.6-1.3) | |
| Andorra | 0.66 (0.45-0.98) | 0.62 (0.38-0.92) | |
| Angola | 1.13 (0.16-3.58) | 0.7 (0.16-2.11) | |
| Antigua and Barbuda | 0.22 (0.2-0.25) | 0.14 (0.12-0.16) | |
| Argentina | 0.04 (0.03-0.05) | 0.09 (0.08-0.1) | |
| Armenia | 0.45 (0.37-0.55) | 0.39 (0.32-0.47) | |
| Australia | 0.18 (0.16-0.2) | 0.36 (0.31-0.42) | |
| Austria | 0.17 (0.15-0.2) | 0.22 (0.19-0.25) | |
| Azerbaijan | 0.51 (0.29-0.81) | 0.55 (0.3-0.94) | |
| Bahamas | 0.33 (0.29-0.38) | 0.36 (0.28-0.45) | |
| Bahrain | 0.3 (0.23-0.42) | 0.2 (0.14-0.28) | |
| Bangladesh | 0.2 (0.14-0.29) | 0.21 (0.13-0.36) | |
| Barbados | 0.2 (0.18-0.22) | 0.18 (0.14-0.24) | |
| Belarus | 0.2 (0.17-0.24) | 0.25 (0.19-0.32) | |
| Belgium | 0.15 (0.13-0.17) | 0.18 (0.15-0.21) | |
| Belize | 0.13 (0.11-0.15) | 0.24 (0.21-0.27) | |
| Benin | 2.46 (1.24-3.93) | 1.69 (1.07-2.4) | |
| Bermuda | 0.21 (0.19-0.23) | 0.11 (0.08-0.13) | |
| Bhutan | 0.29 (0.16-0.47) | 0.37 (0.22-0.6) | |
| Bolivia (Plurinational State of) | 0.3 (0.2-0.41) | 0.24 (0.15-0.38) | |
| Bosnia and Herzegovina | 0.48 (0.4-0.57) | 0.24 (0.17-0.31) | |
| Botswana | 0.67 (0.28-1.41) | 0.95 (0.4-2.27) | |
| Brazil | 0.16 (0.15-0.17) | 0.15 (0.14-0.16) | |
| Brunei Darussalam | 0.84 (0.61-1.18) | 0.6 (0.44-0.82) | |
| Bulgaria | 0.58 (0.44-0.73) | 0.26 (0.19-0.36) | |
| Burkina Faso | 2.92 (1.23-6.59) | 2.46 (1.05-4.75) | |
| Burundi | 0.52 (0.32-0.96) | 0.31 (0.19-0.5) | |
| Cabo Verde | 2.0 (1.4-2.82) | 2.02 (1.4-2.84) | |
| Cambodia | 0.82 (0.44-1.46) | 0.64 (0.3-1.29) | |
| Cameroon | 2.35 (1.63-3.55) | 1.81 (1.02-3.17) | |
| Canada | 0.17 (0.15-0.19) | 0.27 (0.23-0.32) | |
| Central African Republic | 0.99 (0.37-2.09) | 0.63 (0.24-1.4) | |
| Chad | 1.91 (0.74-4.15) | 1.57 (0.84-2.98) | |
| Chile | 0.06 (0.05-0.08) | 0.11 (0.09-0.13) | |
| China | 1.99 (1.66-2.39) | 1.88 (1.47-2.41) | |
| Colombia | 0.22 (0.2-0.24) | 0.15 (0.13-0.19) | |
| Comoros | 0.52 (0.23-0.81) | 0.52 (0.35-0.8) | |
| Congo | 1.08 (0.46-2.14) | 0.71 (0.3-1.5) | |
| Cook Islands | 1.04 (0.71-1.51) | 1.1 (0.71-1.61) | |
| Costa Rica | 0.33 (0.28-0.37) | 0.35 (0.29-0.42) | |
| Croatia | 0.24 (0.18-0.3) | 0.11 (0.08-0.14) | |
| Cuba | 0.18 (0.15-0.21) | 0.11 (0.08-0.13) | |
| Cyprus | 0.15 (0.1-0.21) | 0.13 (0.09-0.17) | |
| Czechia | 0.33 (0.28-0.38) | 0.11 (0.08-0.14) | |
| Cote d'Ivoire | 0.68 (0.41-1.07) | 0.45 (0.26-0.69) | |
| Democratic People's Republic of Korea | 1.86 (0.91-3.31) | 1.46 (0.77-2.72) | |
| Democratic Republic of the Congo | 0.38 (0.19-0.82) | 0.29 (0.13-0.66) | |
| Denmark | 0.1 (0.09-0.12) | 0.06 (0.05-0.07) | |
| Djibouti | 0.34 (0.18-0.6) | 0.44 (0.23-0.75) | |
| Dominica | 0.15 (0.1-0.25) | 0.21 (0.15-0.31) | |
| Dominican Republic | 0.14 (0.1-0.19) | 0.22 (0.15-0.31) | |
| Ecuador | 0.5 (0.43-0.56) | 0.28 (0.21-0.35) | |
| Egypt | 0.8 (0.57-1.15) | 0.85 (0.65-1.11) | |
| El Salvador | 0.13 (0.11-0.15) | 0.13 (0.1-0.16) | |
| Equatorial Guinea | 0.33 (0.16-0.72) | 0.48 (0.23-0.87) | |
| Eritrea | 0.36 (0.22-0.61) | 0.39 (0.22-0.7) | |
| Estonia | 0.32 (0.27-0.38) | 0.23 (0.19-0.27) | |
| Eswatini | 0.98 (0.38-1.91) | 3.46 (1.02-8.88) | |
| Ethiopia | 0.37 (0.24-0.55) | 0.23 (0.15-0.34) | |
| Fiji | 0.49 (0.33-0.71) | 0.58 (0.37-0.86) | |
| Finland | 0.26 (0.22-0.31) | 0.18 (0.15-0.21) | |
| France | 0.26 (0.23-0.28) | 0.28 (0.24-0.32) | |
| Gabon | 0.9 (0.36-2.07) | 0.83 (0.43-1.48) | |
| Gambia | 3.26 (2.16-4.66) | 4.16 (2.56-6.77) | |
| Georgia | 0.4 (0.33-0.46) | 0.23 (0.2-0.27) | |
| Germany | 0.13 (0.12-0.15) | 0.19 (0.17-0.22) | |
| Ghana | 2.41 (1.43-3.88) | 1.7 (1.05-2.78) | |
| Greece | 0.21 (0.2-0.23) | 0.38 (0.35-0.42) | |
| Greenland | 0.57 (0.41-0.79) | 0.35 (0.22-0.52) | |
| Grenada | 0.21 (0.17-0.27) | 0.26 (0.21-0.32) | |
| Guam | 0.32 (0.27-0.4) | 0.94 (0.72-1.17) | |
| Guatemala | 0.53 (0.5-0.56) | 0.4 (0.35-0.46) | |
| Guinea | 3.07 (2.17-4.26) | 2.47 (1.58-3.69) | |
| Guinea-Bissau | 4.35 (1.79-6.99) | 3.03 (1.79-4.54) | |
| Guyana | 0.18 (0.15-0.22) | 0.24 (0.18-0.31) | |
| Haiti | 0.15 (0.08-0.26) | 0.12 (0.06-0.22) | |
| Honduras | 0.15 (0.11-0.21) | 0.12 (0.07-0.2) | |
| Hungary | 0.31 (0.25-0.4) | 0.1 (0.07-0.13) | |
| Iceland | 0.13 (0.11-0.14) | 0.16 (0.13-0.19) | |
| India | 0.2 (0.18-0.24) | 0.27 (0.23-0.33) | |
| Indonesia | 0.37 (0.24-0.54) | 0.43 (0.27-0.68) | |
| Iran (Islamic Republic of) | 0.19 (0.16-0.24) | 0.3 (0.27-0.34) | |
| Iraq | 0.31 (0.22-0.43) | 0.28 (0.2-0.43) | |
| Ireland | 0.09 (0.08-0.11) | 0.12 (0.1-0.14) | |
| Israel | 0.08 (0.07-0.09) | 0.1 (0.09-0.12) | |
| Italy | 0.24 (0.23-0.25) | 0.16 (0.15-0.17) | |
| Jamaica | 0.06 (0.05-0.07) | 0.12 (0.08-0.16) | |
| Japan | 0.43 (0.42-0.44) | 0.18 (0.18-0.19) | |
| Jordan | 0.11 (0.07-0.17) | 0.08 (0.06-0.11) | |
| Kazakhstan | 0.89 (0.79-0.99) | 0.39 (0.33-0.48) | |
| Kenya | 0.23 (0.17-0.34) | 0.37 (0.27-0.54) | |
| Kiribati | 1.06 (0.77-1.49) | 1.1 (0.71-1.65) | |
| Kuwait | 0.29 (0.24-0.34) | 0.06 (0.05-0.07) | |
| Kyrgyzstan | 0.63 (0.47-0.83) | 0.16 (0.11-0.23) | |
| Lao People's Democratic Republic | 1.04 (0.67-1.48) | 0.75 (0.48-1.15) | |
| Latvia | 0.27 (0.22-0.32) | 0.25 (0.19-0.31) | |
| Lebanon | 0.23 (0.16-0.32) | 0.16 (0.12-0.21) | |
| Lesotho | 0.66 (0.21-1.71) | 2.48 (0.81-7.32) | |
| Liberia | 2.4 (1.15-3.91) | 2.43 (1.37-3.76) | |
| Libya | 0.45 (0.31-0.61) | 0.73 (0.5-1.05) | |
| Lithuania | 0.21 (0.18-0.24) | 0.24 (0.2-0.31) | |
| Luxembourg | 0.19 (0.18-0.2) | 0.15 (0.14-0.17) | |
| Madagascar | 0.45 (0.31-0.65) | 0.33 (0.22-0.51) | |
| Malawi | 0.37 (0.24-0.55) | 0.48 (0.32-0.71) | |
| Malaysia | 0.35 (0.27-0.48) | 0.47 (0.35-0.66) | |
| Maldives | 0.38 (0.21-0.61) | 0.36 (0.22-0.55) | |
| Mali | 2.63 (2.02-3.34) | 2.59 (1.8-3.53) | |
| Malta | 0.08 (0.07-0.09) | 0.14 (0.12-0.17) | |
| Marshall Islands | 0.4 (0.25-0.61) | 0.61 (0.36-1.07) | |
| Mauritania | 4.65 (0.95-9.69) | 2.24 (1.09-3.78) | |
| Mauritius | 0.31 (0.28-0.33) | 0.05 (0.05-0.06) | |
| Mexico | 0.12 (0.12-0.12) | 0.17 (0.15-0.19) | |
| Micronesia (Federated States of) | 0.68 (0.44-1.02) | 0.69 (0.39-1.23) | |
| Monaco | 0.27 (0.19-0.4) | 0.55 (0.34-0.88) | |
| Mongolia | 4.17 (2.71-6.13) | 4.62 (3.19-6.6) | |
| Montenegro | 0.45 (0.36-0.56) | 0.38 (0.28-0.5) | |
| Morocco | 0.03 (0.02-0.05) | 0.04 (0.03-0.07) | |
| Mozambique | 0.75 (0.41-1.19) | 1.03 (0.47-2.16) | |
| Myanmar | 0.4 (0.17-0.77) | 0.35 (0.17-0.7) | |
| Namibia | 0.19 (0.1-0.33) | 0.32 (0.19-0.51) | |
| Nauru | 1.1 (0.72-1.6) | 1.01 (0.53-1.68) | |
| Nepal | 0.17 (0.11-0.25) | 0.27 (0.17-0.38) | |
| Netherlands | 0.09 (0.08-0.1) | 0.11 (0.09-0.14) | |
| New Zealand | 0.22 (0.18-0.25) | 0.37 (0.31-0.44) | |
| Nicaragua | 0.2 (0.16-0.24) | 0.21 (0.16-0.27) | |
| Niger | 2.1 (0.92-4.17) | 0.99 (0.59-1.76) | |
| Nigeria | 0.46 (0.19-0.9) | 0.35 (0.2-0.58) | |
| Niue | 0.52 (0.3-0.86) | 0.57 (0.36-0.96) | |
| North Macedonia | 0.62 (0.53-0.75) | 0.38 (0.29-0.48) | |
| Northern Mariana Islands | 0.6 (0.37-0.92) | 0.34 (0.24-0.47) | |
| Norway | 0.14 (0.13-0.14) | 0.29 (0.27-0.32) | |
| Oman | 0.19 (0.11-0.32) | 0.29 (0.19-0.41) | |
| Pakistan | 0.31 (0.23-0.41) | 0.45 (0.33-0.6) | |
| Palau | 0.97 (0.51-1.74) | 1.74 (1.09-2.73) | |
| Palestine | 0.34 (0.23-0.48) | 0.26 (0.2-0.33) | |
| Panama | 0.18 (0.17-0.19) | 0.21 (0.17-0.25) | |
| Papua New Guinea | 0.41 (0.16-1.01) | 0.31 (0.15-0.7) | |
| Paraguay | 0.15 (0.12-0.18) | 0.23 (0.16-0.32) | |
| Peru | 0.24 (0.17-0.34) | 0.25 (0.16-0.36) | |
| Philippines | 1.3 (0.95-1.52) | 0.79 (0.65-0.94) | |
| Poland | 0.05 (0.05-0.06) | 0.12 (0.11-0.13) | |
| Portugal | 0.19 (0.16-0.22) | 0.17 (0.15-0.21) | |
| Puerto Rico | 0.28 (0.23-0.34) | 0.31 (0.25-0.38) | |
| Qatar | 0.46 (0.32-0.71) | 0.5 (0.35-0.69) | |
| Republic of Korea | 2.39 (1.62-3.35) | 0.88 (0.66-1.21) | |
| Republic of Moldova | 0.28 (0.26-0.3) | 0.29 (0.25-0.33) | |
| Romania | 0.15 (0.12-0.18) | 0.15 (0.12-0.19) | |
| Russian Federation | 0.18 (0.18-0.19) | 0.28 (0.26-0.29) | |
| Rwanda | 0.76 (0.5-1.1) | 0.44 (0.27-0.71) | |
| Saint Kitts and Nevis | 0.33 (0.3-0.38) | 0.15 (0.1-0.21) | |
| Saint Lucia | 0.18 (0.15-0.21) | 0.18 (0.15-0.22) | |
| Saint Vincent and the Grenadines | 0.28 (0.24-0.32) | 0.29 (0.25-0.33) | |
| Samoa | 0.36 (0.23-0.55) | 0.41 (0.22-0.65) | |
| San Marino | 0.09 (0.07-0.12) | 0.14 (0.08-0.22) | |
| Sao Tome and Principe | 0.44 (0.28-0.64) | 0.41 (0.21-0.81) | |
| Saudi Arabia | 0.36 (0.23-0.57) | 0.29 (0.18-0.42) | |
| Senegal | 1.75 (0.98-2.66) | 1.18 (0.74-1.74) | |
| Serbia | 0.35 (0.26-0.48) | 0.24 (0.16-0.34) | |
| Seychelles | 0.73 (0.52-0.97) | 0.36 (0.25-0.51) | |
| Sierra Leone | 2.06 (0.65-3.95) | 1.29 (0.81-2.0) | |
| Singapore | 0.6 (0.51-0.69) | 0.22 (0.18-0.27) | |
| Slovakia | 0.41 (0.29-0.57) | 0.24 (0.15-0.37) | |
| Slovenia | 0.39 (0.35-0.45) | 0.16 (0.13-0.19) | |
| Solomon Islands | 0.65 (0.19-1.41) | 0.69 (0.41-1.08) | |
| Somalia | 0.75 (0.32-1.37) | 0.67 (0.3-1.3) | |
| South Africa | 1.16 (0.75-1.74) | 1.24 (1.02-1.49) | |
| South Sudan | 0.44 (0.27-0.71) | 0.57 (0.36-0.89) | |
| Spain | 0.27 (0.24-0.3) | 0.23 (0.19-0.27) | |
| Sri Lanka | 0.19 (0.14-0.25) | 0.13 (0.08-0.19) | |
| Sudan | 0.29 (0.16-0.52) | 0.3 (0.18-0.47) | |
| Suriname | 0.19 (0.12-0.27) | 0.26 (0.17-0.37) | |
| Sweden | 0.2 (0.19-0.22) | 0.2 (0.18-0.24) | |
| Switzerland | 0.28 (0.24-0.33) | 0.12 (0.1-0.14) | |
| Syrian Arab Republic | 0.53 (0.41-0.7) | 0.31 (0.22-0.44) | |
| Taiwan (Province of China) | 1.62 (1.45-1.78) | 1.11 (0.92-1.34) | |
| Tajikistan | 0.57 (0.34-0.89) | 0.4 (0.24-0.61) | |
| Thailand | 1.24 (0.87-1.79) | 1.71 (1.12-2.46) | |
| Timor-Leste | 0.31 (0.17-0.52) | 0.21 (0.11-0.4) | |
| Togo | 1.14 (0.78-1.74) | 1.05 (0.64-1.81) | |
| Tokelau | 0.48 (0.27-0.88) | 0.61 (0.4-0.97) | |
| Tonga | 1.74 (1.14-2.68) | 2.22 (1.32-3.7) | |
| Trinidad and Tobago | 0.19 (0.17-0.21) | 0.26 (0.2-0.34) | |
| Tunisia | 0.12 (0.09-0.16) | 0.18 (0.12-0.26) | |
| Turkmenistan | 0.48 (0.43-0.53) | 0.48 (0.36-0.63) | |
| Tuvalu | 0.6 (0.4-0.92) | 0.62 (0.39-0.96) | |
| Turkey | 0.18 (0.14-0.24) | 0.17 (0.13-0.22) | |
| Uganda | 0.61 (0.4-0.93) | 0.78 (0.52-1.11) | |
| Ukraine | 0.21 (0.18-0.25) | 0.19 (0.13-0.25) | |
| United Arab Emirates | 0.44 (0.28-0.7) | 0.69 (0.44-1.0) | |
| United Kingdom | 0.13 (0.13-0.14) | 0.4 (0.39-0.42) | |
| United Republic of Tanzania | 0.58 (0.39-0.8) | 0.48 (0.31-0.74) | |
| United States Virgin Islands | 0.16 (0.11-0.24) | 0.18 (0.11-0.27) | |
| United States of America | 0.18 (0.18-0.19) | 0.26 (0.25-0.27) | |
| Uruguay | 0.05 (0.04-0.06) | 0.12 (0.1-0.14) | |
| Uzbekistan | 0.34 (0.25-0.45) | 0.44 (0.33-0.57) | |
| Vanuatu | 0.51 (0.28-0.99) | 0.53 (0.31-0.89) | |
| Venezuela (Bolivarian Republic of) | 0.24 (0.23-0.25) | 0.33 (0.25-0.41) | |
| Viet Nam | 1.34 (0.95-1.86) | 1.18 (0.77-1.95) | |
| Yemen | 0.19 (0.06-0.43) | 0.14 (0.06-0.3) | |
| Zambia | 0.98 (0.64-1.56) | 0.47 (0.16-1.32) | |
| Zimbabwe | 1.01 (0.57-1.6) | 1.88 (1.12-2.91) | |
