# Supplementary material for: The descriptive epidemiology and projection of liver cancer in adolescents and young adults: findings from the global burden of disease study 2021
Source: Front Med (Lausanne). 2025 Dec 16;12:1690010. doi: 10.3389/fmed.2025.1690010 (PMC12750614; doi:10.3389/fmed.2025.1690010)
Supplement: Supplementary file 4 [file Table_3.docx]

| **Table S3 Age-standardized** **DALYs rate of liver cancer in youth (15–39 years) by country** | | | |
| --- | --- | --- | --- |
| **Location** | **DALYs in 1990(per 100,000)** | **DALYs in 2021(per 100,000)** |  |
| Afghanistan | 38.54 (26.75-54.39) | 34.43 (24.17-48.96) |  |
| Albania | 50.4 (36.84-65.75) | 30.44 (21.91-42.46) |  |
| Algeria | 7.92 (5.93-10.48) | 10.86 (7.54-15.34) |  |
| American Samoa | 27.22 (19.01-38.26) | 54.23 (35.38-76.35) |  |
| Andorra | 38.78 (26.39-56.2) | 35.89 (22.4-52.6) |  |
| Angola | 66.59 (9.69-209.07) | 41.23 (9.7-125.32) |  |
| Antigua and Barbuda | 12.95 (11.49-14.6) | 8.26 (7.08-9.48) |  |
| Argentina | 2.36 (1.97-2.83) | 5.17 (4.44-5.96) |  |
| Armenia | 26.05 (21.71-32.13) | 22.45 (18.4-26.84) |  |
| Australia | 10.46 (9.39-11.66) | 20.64 (18.03-23.97) |  |
| Austria | 10.12 (9.09-11.45) | 12.84 (11.15-14.51) |  |
| Azerbaijan | 30.05 (17.28-48.03) | 31.95 (17.96-54.76) |  |
| Bahamas | 19.37 (17.08-22.06) | 20.57 (16.14-25.7) |  |
| Bahrain | 17.63 (13.73-24.15) | 11.69 (8.16-15.72) |  |
| Bangladesh | 11.95 (8.5-17.11) | 12.55 (7.67-21.01) |  |
| Barbados | 11.54 (10.42-13.11) | 10.56 (8.26-13.65) |  |
| Belarus | 11.55 (9.69-13.91) | 14.17 (10.79-18.08) |  |
| Belgium | 8.63 (7.64-9.68) | 10.3 (8.88-11.89) |  |
| Belize | 7.89 (6.89-9.27) | 13.98 (12.25-15.78) |  |
| Benin | 145.4 (73.94-232.8) | 99.93 (64.05-140.9) |  |
| Bermuda | 12.15 (10.93-13.41) | 6.07 (4.87-7.51) |  |
| Bhutan | 17.58 (9.77-27.92) | 21.62 (12.64-34.59) |  |
| Bolivia (Plurinational State of) | 17.85 (12.02-24.73) | 14.26 (9.12-22.98) |  |
| Bosnia and Herzegovina | 27.98 (23.54-33.38) | 13.92 (9.76-18.13) |  |
| Botswana | 39.61 (16.67-83.89) | 54.47 (23.19-128.98) |  |
| Brazil | 9.43 (9.06-9.82) | 8.75 (8.3-9.14) |  |
| Brunei Darussalam | 48.6 (35.33-67.96) | 33.76 (24.8-46.19) |  |
| Bulgaria | 33.59 (25.77-41.94) | 14.92 (10.87-20.57) |  |
| Burkina Faso | 172.68 (74.18-386.55) | 144.8 (62.92-278.14) |  |
| Burundi | 31.28 (19.36-56.82) | 18.83 (11.56-30.33) |  |
| Cabo Verde | 119.58 (83.5-170.94) | 117.9 (82.4-163.6) |  |
| Cambodia | 48.32 (25.98-85.89) | 37.15 (17.66-74.86) |  |
| Cameroon | 138.79 (96.48-207.94) | 106.65 (59.67-185.25) |  |
| Canada | 9.75 (8.65-11.02) | 15.98 (13.67-18.41) |  |
| Central African Republic | 58.91 (21.82-124.0) | 37.13 (14.57-82.8) |  |
| Chad | 113.11 (43.84-244.28) | 93.68 (50.32-176.15) |  |
| Chile | 3.6 (2.88-4.45) | 6.08 (5.23-7.25) |  |
| China | 114.52 (95.27-136.94) | 105.85 (83.4-136.14) |  |
| Colombia | 13.3 (12.11-14.54) | 9.16 (7.62-10.95) |  |
| Comoros | 31.62 (13.76-48.59) | 31.22 (21.03-47.87) |  |
| Congo | 63.5 (27.23-128.26) | 41.58 (17.43-87.05) |  |
| Cook Islands | 60.98 (42.09-86.71) | 62.97 (41.26-91.06) |  |
| Costa Rica | 19.14 (16.79-21.53) | 20.69 (17.21-24.89) |  |
| Croatia | 13.8 (10.66-17.39) | 6.24 (4.7-8.24) |  |
| Cuba | 10.54 (9.08-12.3) | 6.22 (4.97-7.66) |  |
| Cyprus | 8.51 (6.03-12.34) | 7.23 (5.11-9.89) |  |
| Czechia | 18.75 (16.21-21.81) | 6.3 (4.79-7.93) |  |
| Cote d'Ivoire | 40.7 (24.46-63.73) | 26.4 (15.42-40.62) |  |
| Democratic People's Republic of Korea | 108.21 (52.88-189.8) | 83.38 (44.49-153.11) |  |
| Democratic Republic of the Congo | 22.69 (11.36-48.8) | 16.96 (7.95-38.85) |  |
| Denmark | 5.94 (5.25-6.67) | 3.71 (3.21-4.3) |  |
| Djibouti | 20.26 (10.82-35.79) | 25.41 (13.55-43.71) |  |
| Dominica | 8.8 (5.76-14.68) | 12.55 (8.73-17.96) |  |
| Dominican Republic | 8.45 (6.23-11.2) | 13.08 (8.86-18.06) |  |
| Ecuador | 30.04 (26.26-33.86) | 16.81 (13.16-21.33) |  |
| Egypt | 47.1 (33.62-67.29) | 49.94 (38.06-64.89) |  |
| El Salvador | 7.87 (6.81-9.21) | 7.76 (6.13-9.72) |  |
| Equatorial Guinea | 19.71 (9.64-41.4) | 28.48 (13.64-51.54) |  |
| Eritrea | 21.55 (13.08-36.04) | 23.08 (13.09-41.72) |  |
| Estonia | 18.49 (15.49-21.7) | 13.39 (11.2-15.86) |  |
| Eswatini | 57.94 (22.48-113.71) | 199.33 (58.99-505.22) |  |
| Ethiopia | 22.15 (14.21-33.04) | 14.02 (9.37-20.84) |  |
| Fiji | 28.7 (19.67-41.7) | 33.53 (21.52-48.87) |  |
| Finland | 14.78 (12.58-17.47) | 10.34 (8.71-12.34) |  |
| France | 14.96 (13.53-16.36) | 16.12 (14.11-18.47) |  |
| Gabon | 52.94 (20.96-119.76) | 48.33 (24.84-85.59) |  |
| Gambia | 192.61 (127.39-273.27) | 246.05 (152.11-400.21) |  |
| Georgia | 22.94 (19.28-26.81) | 13.22 (11.29-15.34) |  |
| Germany | 7.83 (6.83-8.95) | 11.26 (9.75-12.83) |  |
| Ghana | 144.04 (85.31-231.23) | 101.2 (63.16-166.17) |  |
| Greece | 12.81 (11.93-13.69) | 21.82 (20.01-23.86) |  |
| Greenland | 34.29 (24.81-47.24) | 20.44 (12.63-30.12) |  |
| Grenada | 12.61 (9.89-16.17) | 15.12 (12.42-18.66) |  |
| Guam | 18.87 (15.71-23.5) | 53.56 (41.32-66.47) |  |
| Guatemala | 31.63 (29.83-33.49) | 24.16 (20.79-27.44) |  |
| Guinea | 181.12 (126.84-251.49) | 146.27 (93.19-217.67) |  |
| Guinea-Bissau | 257.54 (108.65-413.54) | 178.48 (105.23-267.89) |  |
| Guyana | 10.57 (8.67-13.03) | 14.26 (10.6-18.17) |  |
| Haiti | 8.63 (5.02-15.38) | 6.84 (3.39-13.16) |  |
| Honduras | 9.25 (6.75-12.85) | 7.34 (4.12-11.39) |  |
| Hungary | 17.74 (14.05-22.78) | 5.66 (4.28-7.26) |  |
| Iceland | 7.45 (6.59-8.48) | 9.18 (7.7-11.05) |  |
| India | 11.96 (10.63-13.98) | 15.61 (13.18-19.04) |  |
| Indonesia | 21.61 (13.9-31.53) | 24.55 (15.55-39.03) |  |
| Iran (Islamic Republic of) | 11.74 (9.99-14.47) | 17.6 (15.45-19.8) |  |
| Iraq | 18.31 (13.19-25.27) | 16.87 (11.74-25.55) |  |
| Ireland | 5.54 (4.98-6.16) | 6.87 (5.93-8.05) |  |
| Israel | 4.81 (4.23-5.46) | 5.88 (5.07-6.78) |  |
| Italy | 14.31 (13.68-14.94) | 9.56 (8.99-10.16) |  |
| Jamaica | 3.31 (2.69-4.14) | 6.88 (4.92-9.48) |  |
| Japan | 24.19 (23.6-24.77) | 10.55 (10.24-10.89) |  |
| Jordan | 6.54 (4.36-10.23) | 4.75 (3.31-6.56) |  |
| Kazakhstan | 52.15 (46.73-58.13) | 22.8 (18.83-27.85) |  |
| Kenya | 14.07 (10.12-20.71) | 22.0 (15.66-32.24) |  |
| Kiribati | 63.18 (46.51-87.73) | 64.91 (42.04-98.1) |  |
| Kuwait | 16.78 (14.35-19.81) | 3.44 (2.81-4.3) |  |
| Kyrgyzstan | 36.47 (27.31-48.01) | 9.53 (6.49-13.11) |  |
| Lao People's Democratic Republic | 60.74 (38.97-85.42) | 43.68 (27.9-66.22) |  |
| Latvia | 15.32 (12.7-18.12) | 14.41 (11.36-18.03) |  |
| Lebanon | 13.59 (9.73-18.56) | 9.16 (6.98-12.11) |  |
| Lesotho | 38.8 (12.46-99.8) | 142.67 (47.4-420.02) |  |
| Liberia | 140.04 (67.41-224.14) | 142.68 (80.83-218.79) |  |
| Libya | 26.62 (18.34-36.51) | 42.04 (28.52-60.98) |  |
| Lithuania | 12.25 (10.6-13.99) | 14.18 (11.43-18.01) |  |
| Luxembourg | 11.24 (10.53-11.92) | 8.94 (8.14-9.91) |  |
| Madagascar | 27.41 (19.05-39.14) | 19.8 (12.96-30.99) |  |
| Malawi | 22.2 (14.93-32.89) | 28.88 (18.86-42.62) |  |
| Malaysia | 20.67 (15.97-27.83) | 27.21 (20.23-37.19) |  |
| Maldives | 22.18 (12.35-34.8) | 20.28 (12.38-31.26) |  |
| Mali | 154.89 (119.2-196.81) | 154.51 (108.29-208.6) |  |
| Malta | 4.86 (4.23-5.54) | 8.39 (7.12-10.2) |  |
| Marshall Islands | 23.53 (14.85-36.54) | 35.25 (20.99-60.6) |  |
| Mauritania | 273.85 (56.64-576.86) | 132.56 (64.49-220.89) |  |
| Mauritius | 17.37 (16.07-18.92) | 3.08 (2.78-3.37) |  |
| Mexico | 7.19 (7.05-7.34) | 10.13 (9.12-11.17) |  |
| Micronesia (Federated States of) | 39.67 (25.93-59.24) | 40.25 (23.14-70.88) |  |
| Monaco | 15.86 (10.97-23.07) | 32.34 (19.88-50.55) |  |
| Mongolia | 250.93 (165.2-367.17) | 266.94 (186.28-378.61) |  |
| Montenegro | 26.56 (21.14-33.01) | 21.65 (16.22-28.98) |  |
| Morocco | 2.06 (1.47-2.82) | 2.55 (1.71-4.02) |  |
| Mozambique | 43.81 (24.03-69.15) | 60.53 (28.04-125.29) |  |
| Myanmar | 23.31 (9.92-44.72) | 20.39 (10.04-40.35) |  |
| Namibia | 11.16 (6.18-19.91) | 18.58 (11.12-30.06) |  |
| Nauru | 63.75 (42.09-90.77) | 58.51 (30.69-95.09) |  |
| Nepal | 10.15 (6.83-14.65) | 15.7 (10.27-22.66) |  |
| Netherlands | 5.12 (4.48-6.0) | 6.61 (5.5-7.84) |  |
| New Zealand | 12.74 (10.9-14.92) | 21.53 (18.24-25.63) |  |
| Nicaragua | 11.86 (9.73-14.72) | 12.34 (9.33-15.94) |  |
| Niger | 124.5 (54.98-246.27) | 59.39 (35.24-105.85) |  |
| Nigeria | 27.29 (11.44-53.28) | 20.84 (11.73-34.53) |  |
| Niue | 30.14 (17.45-49.56) | 32.95 (21.2-55.3) |  |
| North Macedonia | 36.33 (30.83-44.01) | 21.82 (16.51-27.56) |  |
| Northern Mariana Islands | 34.3 (21.4-51.95) | 20.13 (14.45-27.21) |  |
| Norway | 7.94 (7.55-8.39) | 17.16 (15.82-18.78) |  |
| Oman | 10.95 (6.68-18.54) | 16.54 (11.06-23.62) |  |
| Pakistan | 18.51 (13.94-24.44) | 26.81 (19.99-36.15) |  |
| Palau | 55.68 (30.08-99.0) | 97.28 (61.77-151.61) |  |
| Palestine | 20.48 (14.04-29.65) | 15.55 (12.15-19.94) |  |
| Panama | 11.07 (10.35-11.66) | 12.4 (10.31-14.75) |  |
| Papua New Guinea | 24.36 (9.5-59.49) | 18.43 (8.81-40.84) |  |
| Paraguay | 8.67 (6.89-10.64) | 13.37 (9.21-18.61) |  |
| Peru | 14.56 (10.43-20.65) | 14.82 (9.9-21.35) |  |
| Philippines | 75.34 (54.95-88.31) | 45.35 (37.55-54.37) |  |
| Poland | 2.94 (2.68-3.28) | 6.73 (6.11-7.43) |  |
| Portugal | 11.16 (9.62-12.88) | 10.07 (8.54-12.3) |  |
| Puerto Rico | 16.36 (13.66-19.74) | 17.88 (14.37-21.82) |  |
| Qatar | 26.61 (18.62-41.01) | 29.28 (20.57-39.94) |  |
| Republic of Korea | 136.0 (92.64-190.16) | 48.9 (36.92-66.87) |  |
| Republic of Moldova | 16.32 (15.3-17.57) | 16.82 (14.64-18.96) |  |
| Romania | 8.64 (6.88-10.51) | 8.94 (7.11-11.24) |  |
| Russian Federation | 10.7 (10.37-11.02) | 15.85 (14.77-16.89) |  |
| Rwanda | 45.83 (29.72-65.16) | 26.28 (16.34-41.62) |  |
| Saint Kitts and Nevis | 19.36 (17.11-22.22) | 8.76 (6.12-12.2) |  |
| Saint Lucia | 10.35 (8.9-12.11) | 10.72 (8.89-12.82) |  |
| Saint Vincent and the Grenadines | 16.31 (14.36-18.83) | 16.66 (14.22-19.12) |  |
| Samoa | 21.4 (13.64-32.2) | 23.83 (12.98-38.53) |  |
| San Marino | 5.31 (4.02-6.95) | 8.15 (4.89-12.57) |  |
| Sao Tome and Principe | 26.11 (16.42-37.95) | 24.24 (12.57-48.34) |  |
| Saudi Arabia | 21.31 (13.37-33.47) | 16.79 (10.8-24.17) |  |
| Senegal | 104.13 (58.48-157.14) | 69.83 (43.89-103.24) |  |
| Serbia | 20.6 (15.08-27.64) | 13.5 (9.31-19.45) |  |
| Seychelles | 41.54 (29.99-55.44) | 20.2 (14.2-28.77) |  |
| Sierra Leone | 121.19 (38.17-233.8) | 76.25 (48.13-118.64) |  |
| Singapore | 34.17 (29.48-39.54) | 12.42 (10.21-15.07) |  |
| Slovakia | 23.18 (16.5-32.21) | 13.86 (8.67-21.08) |  |
| Slovenia | 22.42 (19.91-25.54) | 8.91 (7.28-10.82) |  |
| Solomon Islands | 38.42 (11.51-83.47) | 39.93 (24.39-62.04) |  |
| Somalia | 44.07 (19.0-80.11) | 40.17 (18.2-77.28) |  |
| South Africa | 67.1 (43.66-100.92) | 70.63 (58.3-84.77) |  |
| South Sudan | 26.72 (16.2-42.41) | 34.41 (21.72-53.46) |  |
| Spain | 16.26 (14.61-18.02) | 13.17 (11.3-15.44) |  |
| Sri Lanka | 10.77 (8.09-14.18) | 7.36 (4.57-10.95) |  |
| Sudan | 17.68 (9.84-31.53) | 17.73 (10.73-28.12) |  |
| Suriname | 11.47 (7.18-16.1) | 14.85 (9.97-21.64) |  |
| Sweden | 11.99 (10.97-13.12) | 12.03 (10.48-14.1) |  |
| Switzerland | 16.72 (14.26-19.7) | 6.98 (5.9-8.33) |  |
| Syrian Arab Republic | 31.87 (24.57-42.2) | 18.59 (13.08-25.98) |  |
| Taiwan (Province of China) | 93.26 (84.12-102.02) | 62.01 (51.76-74.53) |  |
| Tajikistan | 33.91 (20.32-53.14) | 23.48 (14.19-36.05) |  |
| Thailand | 72.11 (50.21-103.64) | 96.36 (63.95-136.88) |  |
| Timor-Leste | 18.05 (10.13-30.29) | 12.65 (6.78-23.43) |  |
| Togo | 67.85 (46.39-103.49) | 61.56 (37.99-106.03) |  |
| Tokelau | 28.03 (15.75-51.03) | 35.15 (23.53-55.54) |  |
| Tonga | 101.42 (67.01-155.28) | 127.58 (76.83-208.74) |  |
| Trinidad and Tobago | 10.99 (9.9-12.29) | 15.12 (11.45-19.2) |  |
| Tunisia | 7.11 (5.15-9.64) | 10.29 (6.73-14.76) |  |
| Turkmenistan | 29.04 (26.08-32.15) | 28.05 (20.94-36.84) |  |
| Tuvalu | 34.75 (23.7-53.31) | 36.24 (22.79-55.5) |  |
| Turkey | 10.93 (8.33-14.44) | 9.7 (7.37-12.71) |  |
| Uganda | 36.52 (24.07-56.34) | 46.79 (30.71-66.62) |  |
| Ukraine | 12.62 (10.52-14.97) | 10.96 (7.59-14.41) |  |
| United Arab Emirates | 25.9 (16.43-41.2) | 38.93 (25.06-55.85) |  |
| United Kingdom | 7.89 (7.75-8.05) | 23.51 (22.74-24.38) |  |
| United Republic of Tanzania | 34.57 (24.02-47.98) | 28.87 (18.78-44.23) |  |
| United States Virgin Islands | 9.54 (6.33-14.09) | 10.07 (6.34-15.45) |  |
| United States of America | 10.72 (10.53-10.92) | 15.29 (14.65-15.95) |  |
| Uruguay | 3.04 (2.58-3.61) | 7.09 (6.06-8.31) |  |
| Uzbekistan | 20.53 (15.11-26.71) | 25.55 (19.0-33.29) |  |
| Vanuatu | 30.04 (16.3-57.25) | 31.03 (18.12-51.29) |  |
| Venezuela (Bolivarian Republic of) | 14.24 (13.54-14.95) | 19.47 (15.06-24.7) |  |
| Viet Nam | 77.64 (55.14-107.01) | 67.49 (44.43-109.87) |  |
| Yemen | 10.94 (3.57-25.09) | 8.22 (3.64-17.56) |  |
| Zambia | 59.58 (38.74-95.19) | 27.83 (9.68-78.92) |  |
| Zimbabwe | 59.99 (34.13-93.96) | 111.09 (65.94-171.58) |  |
